# Xanthones Active against Multidrug Resistance and Virulence Mechanisms of Bacteria

**DOI:** 10.3390/antibiotics10050600

**Published:** 2021-05-19

**Authors:** Fernando Durães, Diana I. S. P. Resende, Andreia Palmeira, Nikoletta Szemerédi, Madalena M. M. Pinto, Gabriella Spengler, Emília Sousa

**Affiliations:** 1 Laboratory of Organic and Pharmaceutical Chemistry (LQOF), Department of Chemical Sciences, Faculty of Pharmacy, University of Porto, Rua de Jorge Viterbo Ferreira, 228, 4050-313 Porto, Portugal; fduraes5@gmail.com (F.D.); dresende@ff.up.pt (D.I.S.P.R.); apalmeira@ff.up.pt (A.P.); madalena@ff.up.pt (M.M.M.P.); 2 Interdisciplinary Centre of Marine and Environmental Research (CIIMAR), Terminal de Cruzeiros do Porto de Leixões, Av. General Norton de Matos s/n, 4450-208 Matosinhos, Portugal; 3 Department of Medical Microbiology, Albert Szent-Györgyi Health Center and Faculty of Medicine, University of Szeged, Semmelweis utca 6, 6725 Szeged, Hungary; szemeredi.nikoletta@med.u-szeged.hu

**Keywords:** xanthones, efflux pump, multidrug resistance, antibacterial activity, biofilm inhibition, quorum sensing

## Abstract

The emergence of multidrug and extensively drug-resistant pathogenic bacteria able to resist to the action of a wide range of antibiotics is becoming a growing problem for public health. The search for new compounds with the potential to help in the reversion of bacterial resistance plays an important role in current medicinal chemistry research. Under this scope, bacterial efflux pumps are responsible for the efflux of antimicrobials, and their inhibition could reverse resistance. In this study, the multidrug resistance reversing activity of a series of xanthones was investigated. Firstly, docking studies were performed in the AcrAB-TolC efflux pump and in a homology model of the NorA pump. Then, the effects of twenty xanthone derivatives on bacterial growth were evaluated in *Staphylococcus aureus* 272123 and in the *acrA* gene-inactivated mutant *Salmonella enterica* serovar Typhimurium SL1344 (SE03). Their efflux pump inhibitory properties were assessed using real-time fluorimetry. Assays concerning the activity of these compounds towards the inhibition of biofilm formation and quorum sensing have also been performed. Results showed that a halogenated phenylmethanamine xanthone derivative displayed an interesting profile, as far as efflux pump inhibition and biofilm formation were concerned. To the best of our knowledge, this is the first report of xanthones as potential efflux pump inhibitors.

## 1. Introduction

Currently, drug resistance is rising to dangerously high levels worldwide and threatening our ability to treat even common infectious diseases. Resistance to current anti-infective drug therapies needs to be tackled with additional efforts in industry and scientific research communities in the discovery of new antimicrobial drugs [1]. Multiple antibiotic resistance can arise through a series of distinct molecular mechanisms by different ways: modification of the antibiotic molecule, mutations, modifications and protection of the target, and the prevention of the access of the drug to the target. One example of the latter mechanism is an increase in the efflux of antimicrobials, which can happen through the overexpression of efflux pumps present in the bacterial membrane and lead to multidrug resistance [2]. Efflux pumps are ubiquitous in bacteria and are capable of expelling a multiplicity of compounds from the inside of the bacterial cell, which results in a decrease, or a total lack of efficacy in antimicrobial drugs currently used in the therapy [3]. Therefore, efflux pumps have been regarded as interesting targets for drug development, and many compounds have been described as bacterial efflux pump inhibitors [4], with none of them entering clinical trials up to this date. The mechanisms related to the inhibition of efflux pumps may involve the disruption of the energy supplies of the pumps, membrane destabilization, interaction with components of the pump, or hindrance [5]. As such, the quest for a selective, effective, and non-toxic bacterial efflux pump inhibitor is still open and ongoing [6,7,8,9].

Xanthones are heterocyclic polyphenolic compounds that can be found in microorganisms, fungi, lichens, and some in higher plants and marine sources, with several naturally and synthetically occurring derivatives revealing potent antimicrobial activities over the last few decades [10,11,12]. In recent works, our group described the synthesis of a series of novel nature-inspired chlorinated xanthones [13], and further transformations on the xanthone core in order to achieve a diverse library in terms of molecular function, containing carboxylic acid, ester, methyl, methoxy, phenol, bromo, and amine moieties [14]. The promising results considering their antimicrobial profile, demonstrated mainly by the halogenated and aminated derivatives [14], prompted us to further characterize their antibacterial—and particularly to explore multidrug resistance reversing—activities. Despite their antimicrobial activity, none of these halogenated compounds presented synergy with antimicrobials in resistant bacterial strains [13,14]. On the other hand, a series of hydroxylated xanthones presented synergy with different classes of antimicrobials for the same strains, emphasizing their potential as “antimicrobial adjuvants”, or even as compounds with dual antimicrobial/adjuvant activity [15].

*Salmonella* sp. and *Staphylococcus aureus* are causative agents of infections regarded with high concern both in a clinical setting and in the food industry. These bacteria have not only developed the over-expression of efflux pumps, but also other multiple antibiotic resistance strategies, such as the formation of biofilms, triggered by quorum sensing (QS). Therefore, the search for and development of new compounds that can overcome these mechanisms is urgently necessary [16].

Efflux pumps have also been postulated to be related to other adaptability and virulence mechanisms. In fact, several studies corroborate the link between efflux pumps and the formation of biofilm in Gram-negative bacteria. Specifically, it was shown that the inactivation of genes that code for efflux pumps leads to the formation of defective biofilm or reduce its formation altogether [17,18,19]. While this has not yet been proven for Gram-positive bacteria, it has been shown that efflux pump inhibitors could affect biofilm formation in some Gram-positive bacteria, suggesting a link between efflux pumps and biofilm in these bacteria [20]. QS, the controlled expression of specific genes from bacteria as a response to chemical signals, has a pivotal role in the formation of biofilm and in the expression of virulence factors. Moreover, QS is related to efflux systems, as QS molecules are thought to enter and leave the bacterial cell through these efflux structures [18]. Thus, there is a link between QS and biofilm formation, and compounds effective in both fronts can be candidates for the use in the coating of surfaces, to avoid biofilm formation, or in disinfectants.

Based on previous studies, xanthones are a very important group of compounds to treat microbial infections, being also able to induce apoptosis in tumor cells, inhibit the proliferation of cancer cells and decrease tumor angiogenesis [21]. As highlighted above, efflux pump inhibitors might influence the bacterial virulence interfering with the transport of molecules needed for bacterial communication and biofilm formation. Several xanthone derivatives have shown interest as antimicrobial agents [13,14,15]; however, their mode of action has not been investigated in detail. Herein, in silico and in vitro studies to assess the potential of a substitutional-diverse library of xanthones to inhibit the efflux of an efflux pump substrate in Gram-positive and Gram-negative bacteria are described. Their capability of inhibiting the formation of biofilm, which can also contribute to resistance, was also investigated in Gram-positive bacteria, and their ability to inhibit QS was also evaluated for *Chromobacterium violaceum* and *Serratia marcescens*, strains that inherently have efflux systems of the resistance–nodulation–division (RND) family [17,22,23]. Similar to previous studies [16,24], bacteria in which several efflux systems may be present were chosen as a first screening.

## 2. Results and Discussion

### 2.1. Docking Results

A library of 20 xanthones with diverse substituents was investigated based on preliminary antibacterial activity and synergy studies [13,15,25] against a RND efflux pump model present in Gram-negative bacteria (AcrAB-TolC) and a homology model of the major facilitator superfamily (MFS) efflux pump NorA, prevalent in Gram-positive bacteria. In this study, the used library of xanthones was obtained in-house. The rationale behind this was to test not only xanthones, which presented promising results in antibacterial and/or synergy with antimicrobials, but all the molecular-related xanthones, so that insights into structure–activity relationships could be drawn.

Thus, docking studies were performed in the crystal structure of the AcrB (4DX5), AcrA (2F1M) and TolC (1EK9) portions of the AcrAB-TolC efflux system. For AcrB and AcrA, these studies were performed in two different sites: the substrate-binding site (SBS) and the hydrophobic trap (HT) for AcrB, and the helical hairpin (HH) and the lipoyl domain (LD) for AcrA [26]. For TolC, only the lysine residues that interact with the 3,3′-dithiobis(sulfosuccinimidyl proprionate) (DTSSP) bifunctional crosslinker [26], were considered. For the NorA homology model, the sites used for docking of the compounds were the binding core region (BCR) and the cytoplasmic side (CS), as described in [27]. The results are present in Table 1.

From the docking scores obtained, it can be predicted that the compounds will have increased affinity towards the substrate binding site of the AcrB portion, and less affinity for the hydrophobic trap. The AcrA portion is, in general, the second site with the least favorable docking scores, only better than the hydrophobic trap of AcrB. From these results, a highest affinity towards the AcrB portion can be noted, then for TolC and, lastly, for AcrA. For NorA, an even distribution among compounds is observed with higher affinity towards the binding core region and the cytoplasmic side, hypothesizing that some compounds, such as compounds **1**–**6**, **14**, **18**, and **19**, can act as substrates of the pump, and others, namely compounds **8**–**10**, **12**, **13**, **15**–**17**, and **20** can just block the extracellular efflux by hindering the binding of substrates at a cytoplasmic level. Compounds **7** and **11** revealed similar affinities for both sites. Since these compounds presented docking scores similar to those of compounds already described as inhibitors for the target efflux pumps, in vitro studies were undertaken for this series. It is noteworthy that the controls also showed a better predicted affinity towards the AcrB portion. Recently, reserpine was described as an AcrB inhibitor [28], and the docking study suggested the SBS as one of the binding sites for reserpine. Taking into account the docking results, we chose to study the efflux modulation of a model without the AcrA portion, as this was predicted to be the portion to which the compounds presented the least affinity.

### 2.2. Antibacterial Activity

The compounds were tested for their ability to inhibit bacterial growth in vitro. Results showed that, for the tested strains *Salmonella enterica* serovar Typhimurium 21 SL1344 (SE03) and *Staphylococcus aureus* (*S. aureus*) 272123, none of the investigated compounds displayed antibacterial activity, all showing minimum inhibitory concentrations (MIC) above 100 µM (results not shown). In contrast, compounds **1**, **2**, **11**–**14**, and **18**–**20** exhibited activity for susceptible and resistant bacteria different than the ones present in this study, both Gram-positive and Gram-negative, in previous studies [14,15].

### 2.3. Efflux Pump Inhibition Assay

Compounds **1**–**20** were assessed for their capability of modulating ethidium bromide (EB) accumulation on two resistant strains. *S. aureus* 272123 is a clinical strain and was used to compare the activity of xanthones with natural compounds already tested in the same system; herein, the *mepA* and *norA* genes were studied, and the *norA* expression level did not change [24,29]. These studies suggested that NorA may not be the main pump responsible for efflux in this strain. However, *norA* is a core gene of the *S. aureus* as a species, which means that the NorA pump occurs in all *S. aureus* strains [30].

*Salmonella enterica* serovar Typhimurium SE03, was used as a Gram-negative bacterium. This strain has a deletion of the *acrA* gene, which was predicted with the least affinity for the compounds. The aim of these studies was to perform a first screening of these compounds and their ability to modulate the efflux of EB. All the compounds were tested at the concentration of 50 µM, as none of them showed antibacterial activity at this concentration. The relative fluorescence index (RFI) was calculated based on the means of relative fluorescence units, depicted in Table 2. Reserpine and carbonyl cyanide 3-chlorophenylhydrazone (CCCP) were used as positives control for *S. aureus* 272123 and SE03, respectively, at the sub-MIC concentration of 25 µM.

From the analysis of Table 2, it can be noted that compounds **4**, **5**, **10**–**13**, **16**, **19**, and **20** can increase the fluorescence in comparison to the positive control, which can be attributed to the inhibition of the efflux of EB in the tested bacteria but can also be due to the fluorescence emitted by the compound itself. As such, for these compounds, **4**, **5**, **12**, **13** and **16,** an assay was performed to clarify this matter. In this assay, the compound was tested alone in PBS against a solution of EB, and a solution of EB and the compound together. If the compound presents an irregular fluorescence pattern, or if the fluorescence of the compound with EB is higher than the fluorescence of EB alone, no conclusions can be drawn, as this is a limitation of the assay. The analysis of the curves of the variation of fluorescence over the course of the assay (Supplementary Data, Figures S1–S20) showed that compound **13** presented a descending curve, so it was excluded from further assays. Compound **5** displayed an erratic curve in combination with EB (Supplementary Data, Figures S21–S25), and its results from this assay were not considered. The other tested compounds showed no fluorescence interference in combination with EB. The fact that some compounds presented at least one negative value regarding this assay, implies that the fluorescence of these compounds is lower than that of the control. Therefore, these compounds were deemed as ineffective for the strain where the negative value was obtained.

Five compounds, **4**, **11**, **12**, **19**, and **20**, were able to increase the intracellular fluorescence, attributed to EB, in SE03, while three compounds, **10**, **16**, and **20** could do the same in the *S. aureus* strain tested. For SE03, it can be noted that, in xanthones which were exclusively substituted with hydroxyls, only compound **4** displayed notable activity. This can lead to the conclusion that a hydroxylation in each aromatic ring is required, and even in these specific positions (C-1 and C-7), or at least in the same plane as the ketone. It should be mentioned that compound **4** is a natural product, whose isolation from plants has been described [31,32], and had already demonstrated synergy with antibiotics in Gram-positive and Gram-negative bacteria. Previous results [14] demonstrated that this compound was not active against an extended-spectrum β-lactamase producing strain, emphasizing its selectivity for this particular resistance mechanism.

The introduction of bulkier groups at C-1 led to efflux pump inhibition, as can be seen in the case of **11**, **12**, **19**, and **20**. The presence of methoxyl groups at positions C-3 and C-4 does not seem detrimental for activity, as noted from compounds **8** and **15**. The presence of a methoxyl at position C-6 could be a hindrance for the inhibition of efflux pumps, as none of the compounds with this substituent at this position demonstrated activity in SE03. In the case of compounds **18**–**20**, which are closely related, the fluorine-substituted phenylmethanamine substituent in compound **19** can be highlighted as beneficial, in opposition to a chlorine substitution (**18**). Another noteworthy feature is the presence of a methyl group between the amine and the aromatic ring, as is the case of compound **20**, gifting it with activity. This could reflect a different binding mode from compound **19**, as it presents a chlorine instead of a fluorine and still retains activity. Since the method characterizing the activity of EPIs is working on a real-time basis recording a fluorescent bulk signal, the first step is always the general inhibition of potential efflux systems in bacteria. After this initial screening step, the different mutants lacking efflux pumps genes and strains with overexpressed efflux systems can be investigated. The aim of the present paper was to show the possible targets of xanthones within the bacterial cells, and further investigations with overexpressed pumps are needed.

**Figure 1:**
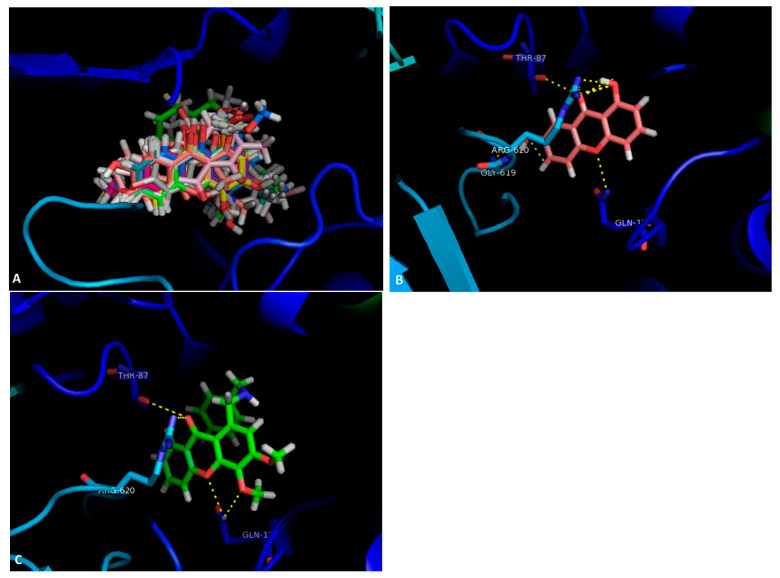
(**A**) Molecular visualization of all the tested compounds in the SBS of AcrB; (**B**) interactions of compound 4 with the SBS; (**C**) interactions of compound 20 with the SBS.

The tested compounds were visualized in PyMol for the SBS of AcrB. It was observed that all the compounds were predicted to bind in approximately the same region (Figure 1A). Compounds **4** and **20** were analyzed in further detail, concerning the residues they interact with. Compound **4** (Figure 1B) can establish a dipole interaction between the carbonyl at C-9 and a carbonyl in Thr-87. The oxygen in the ether moiety forms a hydrogen interaction with a NH_2_ present in Gln-176, and the hydroxyls at C-1 and C-7 interact with Arg-620 and Gly619, respectively.

Compound **19** (Figure 1C) presents the same interactions, apart from the one with Gly-619. The carbonyl at C-9 can form a dipole interaction with a carbonyl in Thr-87 and a hydrogen interaction with a NH_2_ in Arg-620. Furthermore, both the oxygen in the ether moiety and the methoxyl group at C-4 interact with an amide in Gln-176.

The methoxyl groups at C-6 seem to play an important role against *S. aureus* 272123, as compounds **10** and **16** presented these groups not only at this position, but also at positions C-3 and C-4. It also seems that a bulky group at C-1 is an obstacle for the activity, as the trimethoxylated aldehyde **16** presents activity, but a methyl ester does not. The exception to this is compound **20**, which presented one of the best docking scores for the NorA homology model, in the cytoplasmic side. The fact that compound **18** is not an efflux pump inhibitor leads to the conclusion that, once again, the methylene between the amine and the aromatic ring is crucial for this activity.

The data obtained herein do not allow one to establish which efflux pump is being inhibited, only that the efflux of EB is being stopped. Further studies are needed to unequivocally attribute the activity of these compounds to the AcrAB-TolC or NorA efflux pumps, to corroborate docking predictions.

Nonetheless, previous results suggest that these compounds may not act at the level of membrane permeabilization. In fact, these compounds did not show activity in the bacteria used in this study or in other bacteria strains used in previous studies [13,14]. Moreover, when tested against strains that had acquired resistance to antibiotics, many of them did not show synergy with antibiotics [15]. To discharge unspecific effects, the xanthones that were chosen for further assays, **4**, **5**, **11**, **12**, **16**, **19**, and **20**, were screened in SWISSADME [33], and none of the hit compounds showed alerts for pan-assay interference compounds (results not shown). Additionally, some of the oxygenated xanthones presented herein already displayed modulation on P-glycoprotein, a mammalian efflux pump from the ATP-binding cassette family [34]. Despite the fact that no xanthones have yet been described as bacterial efflux pumps, several other related compounds, such as acridones, thioxanthenes, and phenothiazines, have been reported as bacterial ATP-binding cassette and/or MFS inhibitors [35,36]. Phenothiazines were additionally found to interfere with the energy source of the pump [36], which can also be a possible mechanism for xanthones and the decrease in the EB efflux observed. Taking into consideration the results obtained in the efflux pump inhibition assay, compounds **4**, **5**, **11**, **12**, **16**, **19**, and **20**, with favorable results, were selected for deeper studies into other resistance mechanisms, namely biofilm and QS inhibition.

### 2.4. Inhibition of Biofilm Formation

**Figure 2:**
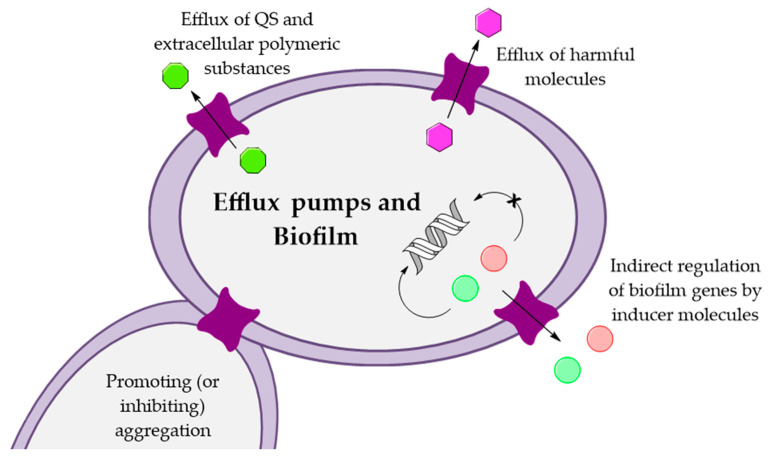
Influence of efflux pumps in biofilm formation mechanisms (adapted from [38]).

Biofilm-mediated tolerance depends on multiple factors, such as slow growth, reduced penetration due to the production of extracellular polysaccharides, and efficient efflux mechanisms [37]. In fact, it has been demonstrated that efflux pumps can influence the transport of QS signal molecules and extracellular polymeric substances in biofilms. Efflux pumps may also regulate the expression of genes involved in biofilm formation. Furthermore, efflux pumps have a crucial role to remove toxic molecules, metabolites, and antibiotics, and they can influence the adhesion and aggregation of bacterial cells to solid surfaces (Figure 2) [38].

Xanthones **4**, **5**, **11**, **12**, **16**, **19**, and **20**, the compounds that presented activity in efflux pumps, were evaluated on their effect on biofilm formation of sensitive and resistant *S. aureus* strains, the first being a reference strain, to compare to the clinical isolate in the EB accumulation assay with overexpressed efflux systems. Compounds **4**, **5**, **11**, **12**, and **19** displayed EB efflux inhibition only in SE03. However, these compounds showed good results in the previous assay, and in order to deepen the insights into their full potential, it was decided to test them in following assays, as they could also interfere in other mechanisms of biofilm formation, adhesion, or degradation. The biofilm inhibition, presented in percentages (%), was calculated based on the mean of absorbance units. Reserpine was used as the control in both strains, as it has been shown to inhibit not only the formation of biofilm in *S. aureus* strains [39], but also bacterial efflux activity [40,41,42]. The results obtained concerning the biofilm inhibition assay are presented in Table 3.

From the results obtained, it can be observed that compounds are overall more effective against *S. aureus* 272123 than the ATCC strain, with only two compounds, **19** and **20**, being effective against the latter. Concerning *S. aureus* 272123, and although only compounds **4**, **19**, and **20** showed higher biofilm inhibition values than reserpine, it can be observed that all the compounds can disrupt this phenomenon to some extent. The most active compound in *S. aureus* 272123 was compound **4**, with over 94% of biofilm formation inhibition compared to the control. This compound did not show a higher RFI than reserpine in the efflux pump assay, leading to the conclusion that a correlation between both assays is difficult to establish for this series of compounds.

The biofilm produced by the ATCC strain was not as influenced by these compounds, except for compounds **19** and **20**; both compounds inhibited over 90% of biofilm formation in this strain. These are the only two compounds tested that possess a halogen in their structure, that could be an important feature for this activity. It is also worth mentioning that compound **20** presented EB inhibition in *S. aureus* 272123, which suggests a possible relationship between efflux pump inhibition and biofilm formation. Further studies are, however, needed to confirm this structure–activity relationship.

### 2.5. Quorum Sensing Assay

The sensor strain *Chromobacterium violaceum* CV026 and the acyl-homoserine lactones (AHLs) producer strain *Sphingomonas paucimobilis* Ezf 10-17 (EZF) were inoculated as parallel lines, and the AHL producers, *Chromobacterium violaceum* wild type 85 (wt85) and *Serratia marcescens* AS-1 were inoculated as a single line. The interaction between the strains and compounds **4**, **5**, **11**, **12**, **16**, **19**, and **20** were evaluated as the reduction in pigment production in millimeters (mm) (Table 4), with promethazine (PMZ) being used as the positive control.

From the analysis of the table, it can be noted that only compounds **12** and **16** inhibited QS in EZF + CV026, evidenced by the discoloration in CV026, which produces a purple pigment due to the QS-dependent expression of the genes that encode the pigment violacein when complemented with an inducing concentration of AHL molecules. Furthermore, compound **12** was an effective efflux pump inhibitor in Gram-negative bacteria; for this reason, this derivative could be a possible candidate for further investigations as an efflux pump inhibitor and QS inhibitor.

### 2.6. Cytotoxicity Assay

In order to apply efflux pump inhibitors to treat patients, several issues should be addressed. Here, three important aspects are highlighted: first, a suitable efflux pump inhibitor must not be antibacterial, because it can lead to resistance; second, the molecule should be selective and not target any eukaryotic efflux pumps; third, it should not be toxic to eukaryotic cells [43]. To assess the toxicity of the best compounds, **4**, **12**, **16**, **19**, and **20**, presenting favorable results in the efflux pump inhibition assay plus on biofilm inhibition and/or QS inhibition assays, a simple toxicity test was carried out on normal mouse fibroblast cells (NIH/3T3). The IC_50_ of the tested compounds is present in Table 5.

The halogenated xanthone **12** and the formylxanthone **16** presented moderate cytotoxicity for the tested cell line. As for the hydroxylated xanthone **4**, no cytotoxicity was observed. The 1-amine halogenated structurally related derivatives **19** and **20** presented very distinct cytotoxicity results, despite behaving similarly in the bacterial studies performed. It can thus be hypothesized that the cytotoxicity from **20** can arise from the chlorine. Aromatic fluorines have attractive features in terms of medicinal chemistry, as these substituents can improve metabolic stability, and decrease the basicity, leading to better bioavailability [44]. Aromatic chlorines have proven, on the other hand, to display toxicity [45]. Overall, it can be noted that the most promising derivatives **19** and **20**, presenting efflux pump inhibition, anti-QS and anti-biofilm properties showed no (IC_50_: >100 µM) and moderate (IC_50_: 35.12 ± 4.86 µM) toxicity, respectively.

## 3. Materials and Methods

### 3.1. Compounds

Xanthones **1**–**3 [46]**, **4** [47], **5** [46,47], **6**–**7** [14], **8**–**10 [13]**, **11** [46,47], **12**–**20** [14] were synthesized as described. The compounds were dissolved in dimethyl sulfoxide (DMSO), for a stock solution of 10 mM to be obtained.

### 3.2. Culture Media and Chemicals

The culture media used in the experiments were the following: cation-adjusted Mueller–Hinton broth (MHB II; Sigma-Aldrich, St. Louis, MO, USA and Biokar Diagnostics, Allone, Beauvais, France), Luria–Bertani broth (LB-B; Sigma, St. Louis, MO, USA), Tryptic Soy broth (TSB; Scharlau Chemie S. A., Barcelona, Spain), and Tryptic Soy agar (TSA; Biokar Diagnostics, Allone, Beauvais, France) were purchased. The modified Luria–Bertani agar (LB*-A), used for the quorum sensing (QS) inhibition assays, was prepared in-house, according to the formula: 1.0 g of yeast extract (Merck, Darmstadt, Germany), 10.0 g of tryptone (Biolab, Budapest, Hungary), 10.0 g of NaCl (Molar Chemicals, Halásztelek, Hungary), 1.0 g of K_2_HPO_4_ (Biolab, Budapest, Hungary), 0.3 g of MgSO_4_∙7H_2_O (Reanal, Budapest, Hungary), 5 mL of Fe-EDTA stock solution and 20.0 g of bacteriological agar (Molar Chemicals, Halásztelek, Hungary) per 1 L of media. *S. aureus* ATCC 29213 was purchased from ATCC and the mouse embryonic fibroblast cell line (NIH/3T3) was purchased from Sigma.

DMSO, 3-(4,5-dimethylthiazol-2-yl)-2,5-diphenyltetrazolium bromide (MTT), sodium dodecyl sulfate (SDS), phosphate-buffered saline (PBS; pH 7.4), EB, reserpine, CCCP, PMZ and crystal violet (CV) were purchased from Sigma-Aldrich Chemie GmbH (Steinheim, Germany). Doxorubicin (2 mg/mL) was purchased from Teva Pharmaceuticals, Budapest, Hungary.

### 3.3. Docking Studies

The crystal structure of the AcrB (PDB: 4DX5) [48], AcrA (PDB: 2F1M) [49], and TolC (PDB: 1EK9) [50] portions of the AcrAB-TolC bacterial efflux system, downloaded from the protein databank (PDB) [51], were used for this study. The known AcrAB-TolC inhibitors D13-9001, doxorubicin, MBX-3132, minocycline, and phenyl-arginyl-β-naphthylamide, along with the tested compounds were drawn with ChemDraw (PerkinElmer Informatics) and minimized using ArgusLab. Docking was carried out using AutoDock Vina (Scripps, CA, USA) [52], in the sites described in [26,53]. The NorA efflux pump does not have an available crystal structure, and a homology model was prepared. The model was generated using the Swiss Model server [54] and the sequence deposited in Uniprot (Q5HHX4) [55], using the EmrD pump from Escherichia coli (PDB: 2GFP) as the homolog, as described in [27]. The top nine poses were collected for each molecule and the lowest docking score value was associated with the most favorable binding conformation. PyMol (Schrödinger) was used for molecular visualization [56].

### 3.4. Bacterial Strains

As Gram-positive bacteria, *Staphylococcus aureus* American Type Culture Collection (ATCC) 29213 and methicillin and ofloxacin-resistant *Staphylococcus aureus* 272123 clinical isolate were used. As Gram-negative bacteria, the acrA gene-inactivated mutant *Salmonella enterica* serovar Typhimurium SL1344 (SE03) was investigated in this study.

For the QS tests, all the bacteria used were Gram-negative. The bacteria used were *Chromobacterium violaceum* wild type 85 (wt85), characterized by the AHL signal molecule-mediated production of the purple violacein pigment, capable of endogenous QS-signal molecule production (N-hexanoyl-l-HSL), *C. violaceum* CV026 (CV026), a Tn5 transposase-mutant, AHL-signal molecule indicator strain (produces purple violacein pigment in the presence of AHL), which is incapable of endogenous QS-signal molecule-production, but useful in the detection of external stimuli, *Sphingomonas paucimobilis* Ezf 10-17 (EZF), AHL-producing-strain (used with *C. violaceum* CV026), and *Serratia marcescens* AS-1, characterized by the AHL signal molecule-mediated production of the orange–red pigment prodigiosin (2-methyl-3-pentyl-6-methoxyprodigiosin), capable of endogenous QS-signal molecule production (N-hexanoyl-l-HSL), were applied [57].

### 3.5. Antibacterial Assay

The antibacterial activity was assessed through the MIC of the compounds. This was determined with the microdilution method, in a 96-well plate, according to the Clinical and Laboratory Standard Institute (CLSI) guidelines [58]. The media used was MHB II. The concentrations tested ranged from 100 µM to 0.195 µM. The MIC was determined by visual inspection. DMSO was used as a solvent for the compounds and was used in subinhibitory concentrations (1% *v*/*v*).

### 3.6. Efflux Pump Inhibition Assay

Compounds **1–20** were evaluated for their ability to inhibit efflux pumps in SE03 and *S. aureus* 272123 strains, through the real-time fluorimetry, monitoring the intracellular accumulation of EB, an efflux pump substrate. This was determined by the automated method using a CLARIOstar Plus plate reader (BMG Labtech, Ortenberg, Germany). Reserpine and CCCP were applied at 25 µM as positive controls, and the solvent DMSO was applied at 1% *v*/*v*. The bacterial strains were incubated in an appropriate culture media (TSB—*S. aureus* 272123; LB-B—SE03) at 37 °C until they reached an optical density (OD) between 0.4 and 0.6 at λ = 600 nm. The culture was centrifuged at 13,000× *g* for 3 min, and the pellet washed and resuspended with PBS. The suspension was centrifuged again in the same conditions and resuspended in PBS. The compounds were applied at 50 µM in a solution of a non-toxic concentration of EB (1 µg/mL) in PBS. Then, 50 µL of this solution were transferred into a 96-well black microtiter plate (Greiner Bio-One Hungary Kft, Mosonmagyaróvár, Fertősor, Hungary), and 50 µL of bacterial suspension (OD_600_ 0.4–0.6) were added to each well. The plates were placed into the CLARIOstar plate reader, and the fluorescence was monitored at excitation and emission wavelengths of 530 nm and 600 nm every minute for one hour on a real-time basis. From the real-time data, the activity of the compounds, namely the RFI of the last time point (minute 60) of the EB accumulation assay, was calculated according to the following formula:

(1) $$\mathrm{RFI}=\frac{{\mathrm{RF}}_{\mathrm{treated}}-{\mathrm{RF}}_{\mathrm{untreated}}}{{\mathrm{RF}}_{\mathrm{untreated}}}$$

where RF_treated_ is the relative fluorescence (RF) at the last time point of EB accumulation curve in the presence of the compound, and RF_untreated_ is the RF at the last time point of the EB accumulation curve of the untreated control, having only the solvent (DMSO) control. The accumulation curves were designed using Microsoft Excel^®^.

### 3.7. Inhibition of Biofilm Formation

The derivatives **4**, **5**, **11**, **12**, **16**, **19**, and **20** were tested for their ability to decrease the formation of biofilm. The bacterial strains used were the Gram-positive *S. aureus* ATCC 25923 and *S. aureus* 272123. The detection of the biofilm formation was possible with the use of the dye crystal violet (CV; 0.1% *v/v*). The initial inoculum was incubated in TSB overnight, and then diluted to an OD_600_ of 0.1. Then, the bacterial suspension was added to 96-well microtiter plates and the compounds were added at half the MIC. If the MIC was >100 µM, the compound would be added at the concentration of 100 µM. The final volume in each well was 200 µL. Reserpine was used as the positive control, as it was the same compound used in the efflux pump inhibition assay and it has shown activity in the inhibition of biofilm formation in *S. aureus* strains [39]. The plates were incubated at 30 °C for 48 h, with gentle stirring (100 rpm). After this incubation period, the TSB medium was discarded, and the plates were washed with tap water to remove unattached cells. Afterwards, 200 µL of a 0.1% *v/v* CV solution were added to the wells and incubated for 15 min at room temperature. Then, the CV solution was removed from the wells, and the plates were washed again with tap water, and 200 µL of a 70% ethanolic solution were added to the wells. The biofilm formation was determined by measuring the OD_600_ using a Multiscan EX ELISA plate reader (Thermo Labsystems, Cheshire, WA, USA). The anti-biofilm effect of the compounds was expressed as the percentage (%) of decrease in biofilm formation.

### 3.8. Quorum Sensing Assay

The QS inhibitory effect of the compounds was examined on the EZF and the sensor CV026 strains, on the wt85 strain, and on *S. marcescens*, for **4**, **5**, **11**, **12**, **16**, **19**, and **20**. The method used was the parallel inoculation method, where pair combinations of the used sensor strain CV026 and the *N*-acyl-homoserine lactone (AHL)-producing strain EZF were inoculated directly onto the LB*-A agar surface in parallel, at an approximate distance of 5 mm from each other. *S. marcescens* AS-1 and wt85 were inoculated as a single line. Filter paper disks (7 mm in diameter) were placed on the center of the inoculated line(s) and impregnated with 8 µL of a solution of 10 mM of the compounds. PMZ was used as the positive control, as previous results have demonstrated its activity as a QS inhibitor [59]. The agar plates were incubated at room temperature (20 °C) for 24–48 h. The QS inhibition was accessed visually, through the inhibition of pigment production. The discolored, but intact, bacterial colonies were measured with a ruler [16,59].

### 3.9. Cytotoxicity Assay

Mouse fibroblasts (NIH/3T3, ATCC CRL-1658TM) were cultivated in DMEM (Gibco 52100-039) and supplemented with 10% heat-inactivated fetal bovine serum (Biowest, VWR International Kft, Debrecen, Hungary), 2 mM of l-glutamine, 1 mM Na pyruvate, 100 U/L and 10 mg/L penicillin/streptomycin mixture (Sigma-Aldrich Chemie GmbH, Steinheim, Germany), respectively, and 0.1% nystatin (8.3 g/L in ethylene glycol). The adherent cells were detached using a combination of 0.25% Trypsin–Versene (EDTA) solution for 5 min at 37 °C. Before each cytotoxicity assay using this cell line, cells were seeded in untreated 96-well flat-bottom microtiter plates, following a 4-h incubation period in a humidified atmosphere (5% CO_2_, 95% air) at 37 °C [60].

The cytotoxicity of **4**, **12**, **16**, **19**, and **20** was assessed in NIH/3T3 cells. The compounds were added to the cells distributed into 96-well flat bottom microtiter plates at concentrations of 1 × 10^4^ and initially incubated for 24 h, after which a solution of MTT in PBS was added to each well and incubated for another 4 h. The concentrations used were the same as in the MIC assay. After this, 100 μL of SDS (10% in a 0.01 M HCl solution) were added to each well and incubated overnight at 37 °C. Doxorubicin was used as the positive control. Cell growth was determined in quadruplicate by measuring OD at λ = 540 nm (reference 630 nm) in a Multiscan EX ELISA reader (Thermo Labsystems, Cheshire, WA, USA). The percentage of inhibition of cell growth was determined according to the equation:

(2) $$100-\left(\frac{{\mathrm{OD}}_{\mathrm{sample}}-{\mathrm{OD}}_{\mathrm{medium}\;\mathrm{control}}}{{\mathrm{OD}}_{\mathrm{cell}\;\mathrm{control}}-{\mathrm{OD}}_{\mathrm{medium}\;\mathrm{control}}}\right)\times 100$$

The results were expressed as the mean ± standard deviation (SD), and the IC_50_ values were obtained by best fitting the dose-dependent inhibition curves in GraphPad Prism 5.03 for Windows software.

## 4. Conclusions

In the present study, various efflux pump-related aspects of bacterial resistance have been investigated. Since it is the first report on xanthones as potential EPIs, the general activity of the compounds and the interplay with different virulence and resistance determinants were investigated (bacterial communication, biofilm formation, efflux pump inhibition). The results herein disclosed the potential of xanthone derivatives to circumvent antimicrobial resistance mechanisms. Although none of the tested compounds displayed antibacterial activity for both tested strains, in the range of concentrations used, compounds **4**, **10**, **11**, **12**, **16**, **19**, and **20** were effective at decreasing the efflux of EB in the tested strains, which can translate to the inhibition of efflux pumps. These results are corroborated by the docking studies performed, that showed that these compounds present good scores for this target. Concerning the biofilm formation assay, compounds **4**, **5**, **11** and **12** are more active in *S. aureus* 272123 than in the ATCC, even though only **4** presents an inhibition of biofilm formation superior to the positive control. Compounds **19** and **20** were more effective than reserpine at inhibiting this phenomenon in both tested strains, an effect more noted with the ATCC strain. In fact, these compounds were also able to decrease biomass in the ATCC strain, with results of 97% and 91%, respectively. Noteworthy is also the fact both these compounds, **19** and **20**, presented promising results on the inhibition of efflux pumps. Concerning QS, two compounds, **12** and **16**, were able to inhibit this virulence mechanism in the combination of EZF and CV026. Finally, two derivatives, **4** and **19**, arise as hit compounds, for their overall results against bacteria resistance and virulent mechanisms and their lack of cytotoxicity, with potential to be used as safe antibiotic adjuvants in the treatment of skin infections.

The overall results show xanthone derivatives are effective in inhibiting efflux pumps, but also present efficacy against other resistance mechanisms with different hits found in different experiments. To the best of our knowledge, this is the first time xanthone derivatives have been described as bacterial efflux pump inhibitors. Future studies may bring insight into the synergy possibilities of hit compound **19** with other halogenated xanthones herein described, such as compound **18**, whose antibacterial activity has been described for Gram-negative bacteria, but displayed no activity as a bacterial efflux pump inhibitor. Even though their results in the docking studies suggest they could interact with efflux structures, the underlying mechanisms must also be studied in a deeper extent, to overcome limitations of the real-time EB accumulation assay. Genetic assays, for instance, are warranted to unequivocally attribute the activity of these compounds to a specific efflux pump, such as AcrAB-TolC or NorA, since they may act through different mechanisms or inhibit different efflux systems.

## Figures and Tables

**Table 1:** Structures of the xanthone derivatives and docking results for the compounds.

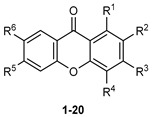													
Compound							Docking Score						
							AcrB		AcrA		TolC	NorA	
No.	R^1^	R^2^	R^3^	R^4^	R^5^	R^6^	SBS	HT	HH	LD		BCR	CS
**1**	H	H	H	OH	H	H	−6.7	−5.9	−6.2	−6.2	−6.4	−6.6	−6.3
**2**	H	OH	OH	H	H	H	−6.9	−6.2	−6.2	−6.7	−6.5	−6.2	−6.1
**3**	OH	OH	H	H	H	H	−6.6	−5.5	−6.4	−6.8	−7.0	−6.5	−6.4
**4**	OH	H	H	H	H	OH	−6.9	−5.9	−6.4	−6.7	−6.5	−6.6	−6.3
**5**	OH	CH_3_	OH	H	H	H	−6.9	−6.2	−6.4	−6.3	−6.6	−6.2	−5.5
**6**	CH_3_	H	OH	OH	H	H	−6.9	−6.1	−6.7	−7.0	−6.7	−6.9	−6.6
**7**	CH_3_	H	OH	OH	OH	H	−7.2	−6.4	−6.0	−7.8	−6.6	−5.9	−5.9
**8**	CH_3_	H	OCH_3_	OCH_3_	H	H	−6.8	−5.9	−5.7	−5.9	−6.6	−5.1	−5.4
**9**	CH_3_	Cl	OCH_3_	OCH_3_	H	H	−7.0	−5.1	−5.8	−5.9	−6.4	−4.8	−5.2
**10**	CH_3_	H	OCH_3_	OCH_3_	OCH_3_	H	−7.1	−5.5	−5.3	−6.1	−6.6	−4.8	−5.2
**11**	OCH_3_	H	H	H	H	H	−6.8	−5.1	−6.4	−6.0	−6.7	−6.8	−6.8
**12**	CH_2_Br	H	OCH_3_	OCH_3_	H	H	−7.1	−5.0	−5.3	−5.5	−6.2	−3.4	−5.7
**13**	CH_2_Br	H	OCH_3_	OCH_3_	OCH_3_	H	−7.0	−5.0	−5.1	−5.7	−6.1	−2.9	−5.6
**14**	CHO	H	OCH_3_	OH	H	H	−7.2	−6.2	−5.7	−6.7	−6.9	−6.3	−5.3
**15**	CHO	H	OCH_3_	OCH_3_	H	H	−7.2	−5.0	−5.5	−6.1	−6.2	−4.7	−5.2
**16**	CHO	H	OCH_3_	OCH_3_	OCH_3_	H	−7.3	−5.9	−5.3	−6.4	−6.3	−4.7	−5.1
**17**	CO_2_CH_3_	H	OCH_3_	OCH_3_	OCH_3_	H	−7.5	−5.9	−5.5	−4.9	−6.5	−4.5	−5.2
**18**	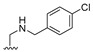	H	OCH_3_	OCH_3_	H	H	−8.0	8.6	−7.0	−6.2	−7.9	−7.9	−5.4
**19**	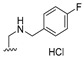	H	OCH_3_	OCH_3_	H	H	−7.3	−5.8	−6.3	−6.3	−6.9	−7.9	−6.0
**20**	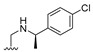	H	OCH_3_	OCH_3_	H	H	−7.6	−5.5	−7.0	−6.4	−7.9	−5.6	−6.4
**D13-9001**							−9.7	26.5	−6.2	−5.1	−7.4	0.7	−4.7
**Doxorubicin**							−8.9	15.4	−7.2	−5.6	−7.2	−0.5	−4.7
**MBX-3132**							−7.9	2.9	−7.9	−6.2	−7.7	−6.2	−7.0
**Minocycline**							−8.7	26.7	−6.2	−5.4	−7.7	1.3	−4.9
**PAβN**							−7.1	−4.7	−5.8	−4.9	−7.1	−9.4	−5.3
**Reserpine**							−8.7	10.9	5.6	4.6	−7.5	1.0	−4.6

**Table 2:** Relative fluorescence index of tested derivatives. Compounds were tested in the same conditions, on different assays (five for *S. aureus* and six for SE03), and the superscript numbers are relative to the positive control obtained in each assay.

RFI ± SD		
Compound	*S. aureus* 272123	SE03
**1**	−0.36 ± 0.03 ^1^	−0.01 ± 0.05 ^6^
**2**	−0.69 ± 0.02 ^2^	−0.57 ± 0.02 ^7^
**3**	−0.05 ± 0.06 ^3^	0.23 ± 0.04 ^8^
**4**	0.13 ± 0.08 ^2^	2.90 ± 0.71 ^7^
**5**	0.16 ± 0.08 ^2^	1.74 ± 0.22 ^7^
**6**	−0.91 ± 0.01 ^4^	−0.91 ± 0.01 ^9^
**7**	−0.92 ± 0.00 ^5^	−0.87 ± 0.02 ^10^
**8**	0.15 ± 0.25 ^4^	0.05 ± 0.11 ^9^
**9**	−0.04 ± 0.26 ^4^	0.05 ± 0.09 ^9^
**10**	0.18 ± 0.20 ^5^	0.24 ± 0.05 ^10^
**11**	−0.10 ± 0.06 ^1^	0.28 ± 0.09 ^6^
**12**	0.11 ± 0.30 ^4^	1.75 ± 1.57 ^9^
**13**	0.53 ± 0.20 ^5^	1.16 ± 0.64 ^10^
**14**	−0.28 ± 0.06 ^2^	0.02 ± 0.02 ^7^
**15**	0.08 ± 0.07 ^4^	0.13 ± 0.09 ^9^
**16**	5.49 ± 8.04 ^5^	0.13 ± 0.15 ^10^
**17**	−0.34 ± 0.02 ^5^	−0.29 ± 0.08 ^10^
**18**	−0.01 ± 0.04 ^3^	0.04 ± 0.04 ^8^
**19**	0.02 ± 0.02 ^3^	2.86 ± 0.14 ^8^
**20**	1.08 ± 0.82 ^4^	2.09 ± 0.05 ^11^
Reserpine	^1^ 0.31 ± 0.07 ^2^ 0.45 ± 0.04 ^3^ 0.84 ± 0.13 ^4^ 0.35 ± 0.14 ^5^ 0.16 ± 0.05	---
CCCP	---	^6^ 0.23 ± 0.04 ^7^ 0.37 ± 0.04 ^8^ 0.33 ± 0.09 ^9^ 0.40 ± 0.03 ^10^ 0.27 ± 0.14 ^11^ 0.50 ± 0.11

^1–11^ The value of the positive control in each different assay. SD: standard deviation.

**Table 3:** Percentage of biofilm inhibition of the selected compounds. The compounds were tested in the same conditions, on two different assays for each strain, and the superscript numbers are relative to the positive control obtained in each assay.

Inhibition of Biofilm Formation (%) ± SD		
Compound	*S. aureus* ATCC 29213	*S. aureus* 272123
**4**	0 ^1^	94.21 ± 1.31 ^3^
**5**	0 ^1^	61.62 ± 16.51 ^3^
**11**	0 ^1^	39.95 ± 7.66 ^3^
**12**	0 ^1^	6.27 ± 1.41 ^3^
**16**	0 ^2^	58.98 ± 12.00 ^4^
**19**	97.45 ± 0.62 ^2^	65.03 ± 9.86 ^4^
**20**	90.76 ± 1.62 ^2^	77.17 ± 4.45 ^4^
Reserpine	^1^ 2.49 ± 1.99 ^2^ 22.29 ± 10.88	^3^ 77.62 ± 10.44 ^4^ 63.42 ± 2.63

^1–4^ The value of the positive control in each different assay. SD: standard deviation.

**Table 4:** Results of the quorum sensing inhibition assay.

Quorum Sensing Inhibition (mm) ± SD			
Compound	*S. marcescens*	EZF + CV026	wt85
**4**	0	0	0
**5**	0	0	0
**11**	0	0	0
**12**	0	30 ± 0.5	0
**16**	0	42 ± 0.8	0
**19**	0	0	0
**20**	0	0	0
PMZ	18 ± 0.8	40 ± 0.1	41 ± 0.5

SD: standard deviation.

**Table 5:** Cytotoxicity (IC_50_) of the tested compounds.

Compound	IC_50_ (µM) ± SD
**4**	>100
**12**	54.59 ± 5.30
**16**	26.93 ± 5.87
**19**	>100
**20**	35.12 ± 4.86
Doxorubicin	12.05 ± 0.81

## Data Availability

The data presented in this study are available in the article and in the Supplementary Materials.

## Supplementary Materials

The following are available online at https://www.mdpi.com/article/10.3390/antibiotics10050600/s1, Figure S1: Fluorescence curves for the EB accumulation assay for compound **1**, Figure S2: Fluorescence curves for the EB accumulation assay for compound **2**, Figure S3: Fluorescence curves for the EB accumulation assay for compound **3**, Figure S4: Fluorescence curves for the EB accumulation assay for compound **4**, Figure S5: Fluorescence curves for the EB accumulation assay for compound **5**, Figure S6: Fluorescence curves for the EB accumulation assay for compound **6**, Figure S7: Fluorescence curves for the EB accumulation assay for compound **7**, Figure S8: Fluorescence curves for the EB accumulation assay for compound **8**, Figure S9: Fluorescence curves for the EB accumulation assay for compound **9**, Figure S10: Fluorescence curves for the EB accumulation assay for compound **10**, Figure S11: Fluorescence curves for the EB accumulation assay for compound **11**, Figure S12: Fluorescence curves for the EB accumulation assay for compound **12**, Figure S13: Fluorescence curves for the EB accumulation assay for compound **13**, Figure S14: Fluorescence curves for the EB accumulation assay for compound **14**, Figure S15: Fluorescence curves for the EB accumulation assay for compound **15**, Figure S16: Fluorescence curves for the EB accumulation assay for compound **16**, Figure S17: Fluorescence curves for the EB accumulation assay for compound **17**, Figure S18: Fluorescence curves for the EB accumulation assay for compound **18**, Figure S19: Fluorescence curves for the EB accumulation assay for compound **19**, Figure S20: Fluorescence curves for the EB accumulation assay for compound **20**, Figure S21: Fluorescence curves for compound **4**, Figure S22: Fluorescence curves for compound **5**, Figure S23: Fluorescence curves for compound **12**, Figure S24: Fluorescence curves for compound **13**, Figure S25: Fluorescence curves for compound **16**.

## Abbreviations

**AHL**	Acyl-homoserine-lactone
**ATCC**	American Type Culture Collection
**BCR**	Binding core region
**CCCP**	Carbonyl cyanide 3-chlorophenylhydrazone
**CS**	Cytoplasmic side
**CV026**	*Chromobacterium violaceum* CV026
**CV**	Crystal violet
**DMSO**	Dimethyl sulfoxide
**DTSSP**	3,3′-dithiobis(sulfosuccinimidyl propionate)
**EB**	Ethidium bromide
**EZF**	*Sphingomonas paucimobilis* EZF 10-17
**HH**	Helical hairpin
**HT**	Hydrophobic trap
**IC_50_**	Half-maximal inhibitory concentration
**LB-A**	Luria–Bertani agar
**LB-B**	Luria–Bertani broth
**LD**	Lipoyl domain
**MFS**	Major facilitator superfamily
**MHB II**	Cation-adjusted Mueller–Hinton broth
**MIC**	Minimum inhibitory concentration
**MTT**	3-(4,5-dimethylthiazol-2-yl)-2,5-diphenyltetrazolium bromide
**OD**	Optical density
**PBS**	Phosphate-buffered saline
**PMZ**	Prometazine
**RF**	Relative fluorescence
**RFI**	Relative fluorescence index
**RND**	Resistance-nodulation-division
**SBS**	Substrate-binding site
**SD**	Standard deviation
**SE03**	*Salmonella enterica* serovar Typhimurium with the *acrA* gene deleted
**TSA**	Tryptic Soy agar
**TSB**	Tryptic Soy broth
**QS**	Quorum sensing
**wt85**	*Chromobacterium violaceum* wild type 85
