# Supplementary material for: Xanthones Active against Multidrug Resistance and Virulence Mechanisms of Bacteria
Source: Antibiotics (Basel). 2021 May 19;10(5):600. doi: 10.3390/antibiotics10050600 (PMC8158687; doi:10.3390/antibiotics10050600)
Supplement: Supplementary file 1 [file antibiotics-10-00600-s001.zip › antibiotics-1188512-supplementary.pdf]

Supplementary Materials

Xanthones active against multidrug resistance and virulence mechanisms of bacteria

Fernando Durães [1,2], Diana I. S. P. Resende [1,2], Andreia Palmeira [1,2], Nikoletta Szemerédi [3], Madalena M. M. Pinto [1,2], Gabriella Spengler [3,*], Emília Sousa [1,2,*]

1 Laboratory of Organic and Pharmaceutical Chemistry (LQOF), Department of Chemical Sciences, Faculty of Pharmacy, University of Porto, Rua de Jorge Viterbo Ferreira, 228, 4050-313 Porto, Portugal; fduraes5@gmail.com (F. D); apalmeira@ff.up.pt (A.P.).

2 Interdisciplinary Centre of Marine and Environmental Research (CIIMAR), Terminal de Cruzeiros do Porto de Leixões, Av. General Norton de Matos s/n, 4450-208 Matosinhos, Portugal; dresende@ff.up.pt (D. I. S. P. R.); madalena@ff.up.pt (M. P.).

3 Department of Medical Microbiology and Immunobiology, Faculty of Medicine, University of Szeged, Dóm tér 10, 6720 Szeged, Hungary; szemeredi.nikoletta@med.u-szeged.hu (N. S.).

* Correspondence: spengler.gabriella@med.u-szeged.hu (G. S.); esousa@ff.up.pt (E. S.).

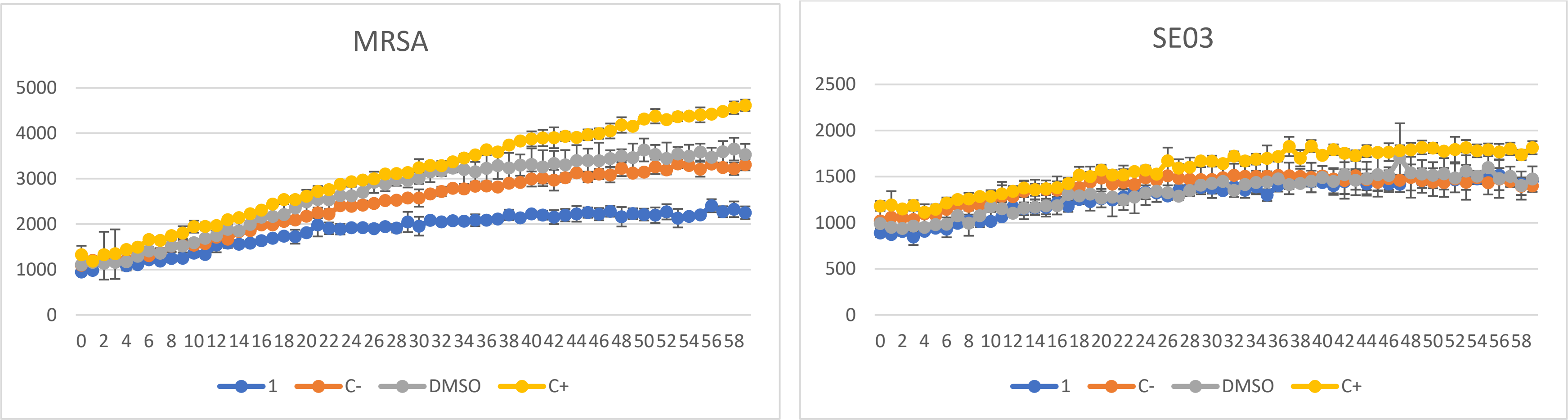

Figure S1. Fluorescence curves for the EB accumulation assay for compound **1**. Conditions: **1** – 50 µM of compound **1** in a solution of EB in PBS (1 µg/mL); **C-** – Bacteria in a solution of EB in PBS (1 µg/mL); **C+** – 25 µM of reserpine in a solution of EB in PBS (1 µg/mL); **DMSO** – 1% v/v of DMSO in a solution of EB in PBS (1 µg/mL)

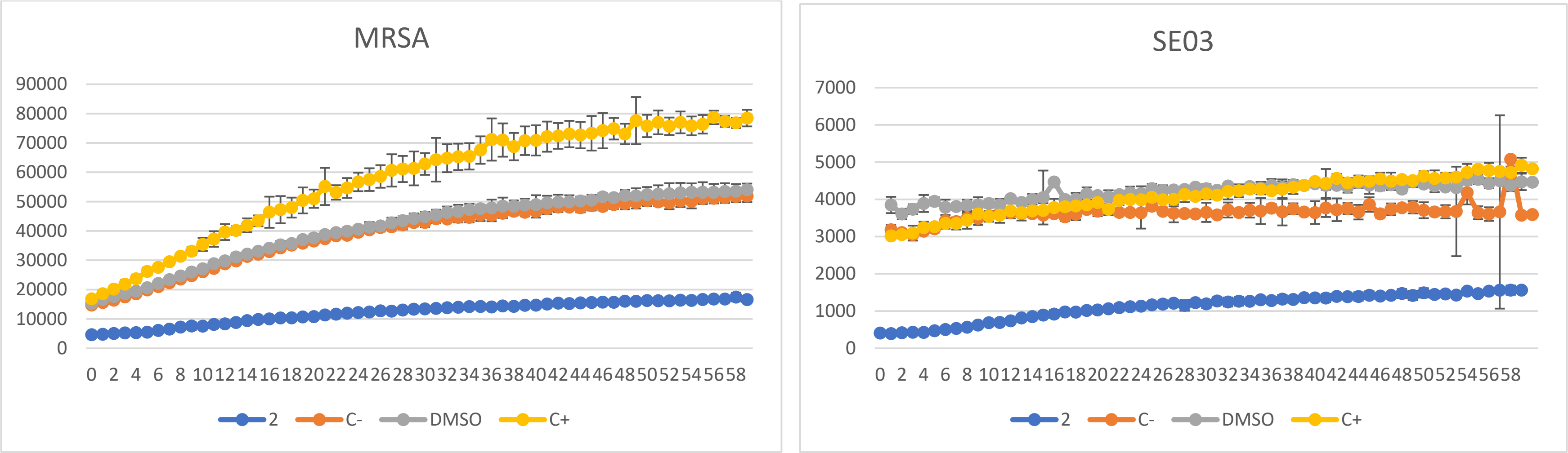

Figure S2. Fluorescence curves for the EB accumulation assay for compound **2**. Conditions: **2** – 50 μM of compound **2** in a solution of EB in PBS (1 μg/mL); **C-** – Bacteria in a solution of EB in PBS (1 μg/mL); **C+** – 25 μM of reserpine in a solution of EB in PBS (1 μg/mL); **DMSO** – 1% v/v of DMSO in a solution of EB in PBS (1 μg/mL)

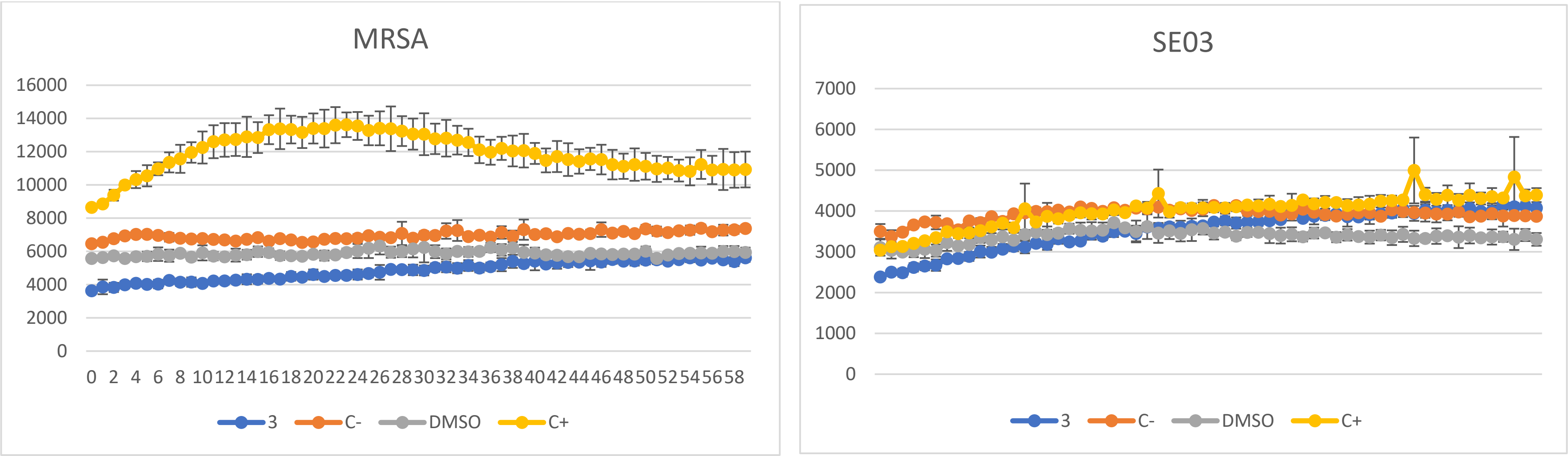

Figure S3. Fluorescence curves for the EB accumulation assay for compound **3**. Conditions: **3** – 50 μM of compound **3** in a solution of EB in PBS (1 μg/mL); **C-** – Bacteria in a solution of EB in PBS (1 μg/mL); **C+** – 25 μM of reserpine in a solution of EB in PBS (1 μg/mL); **DMSO** – 1% v/v of DMSO in a solution of EB in PBS (1 μg/mL)

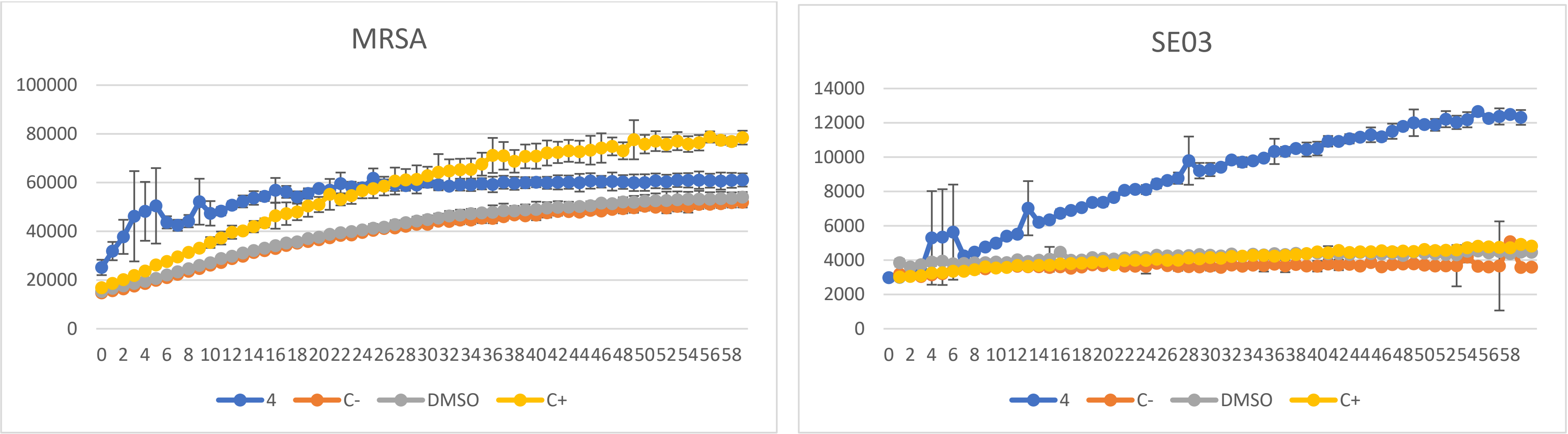

Figure S4. Fluorescence curves for the EB accumulation assay for compound **4**. Conditions: **4** – 50 µM of compound **4** in a solution of EB in PBS (1 µg/mL); **C-** – Bacteria in a solution of EB in PBS (1 µg/mL); **C+** – 25 µM of reserpine in a solution of EB in PBS (1 µg/mL); **DMSO** – 1% v/v of DMSO in a solution of EB in PBS (1 µg/mL)

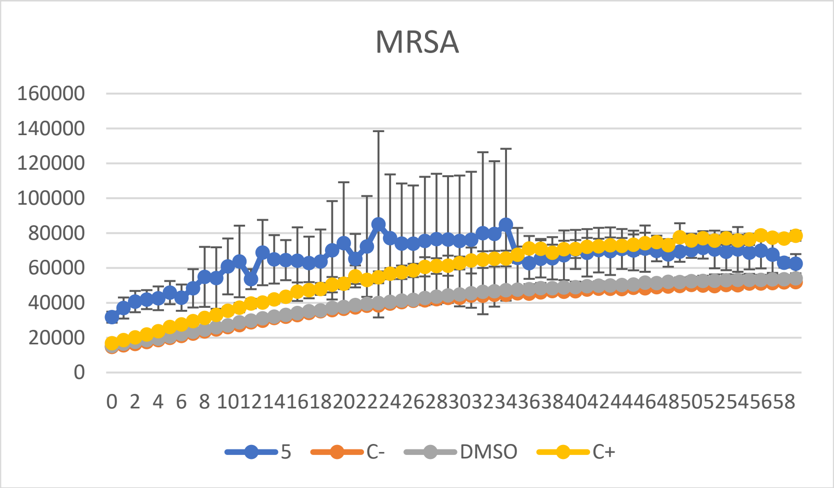

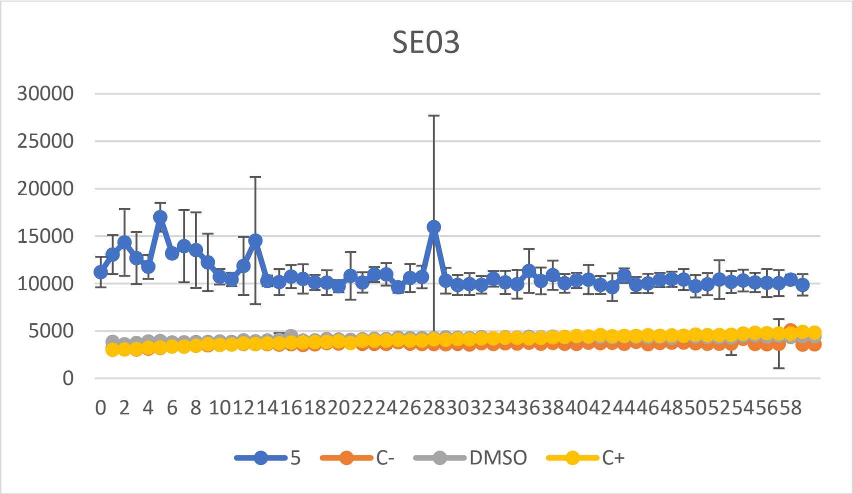

Figure S5. Fluorescence curves for the EB accumulation assay for compound **5**. Conditions: **5** – 50 µM of compound **5** in a solution of EB in PBS (1 µg/mL); **C-** – Bacteria in a solution of EB in PBS (1 µg/mL); **C+** – 25 µM of reserpine in a solution of EB in PBS (1 µg/mL); **DMSO** – 1% v/v of DMSO in a solution of EB in PBS (1 µg/mL)

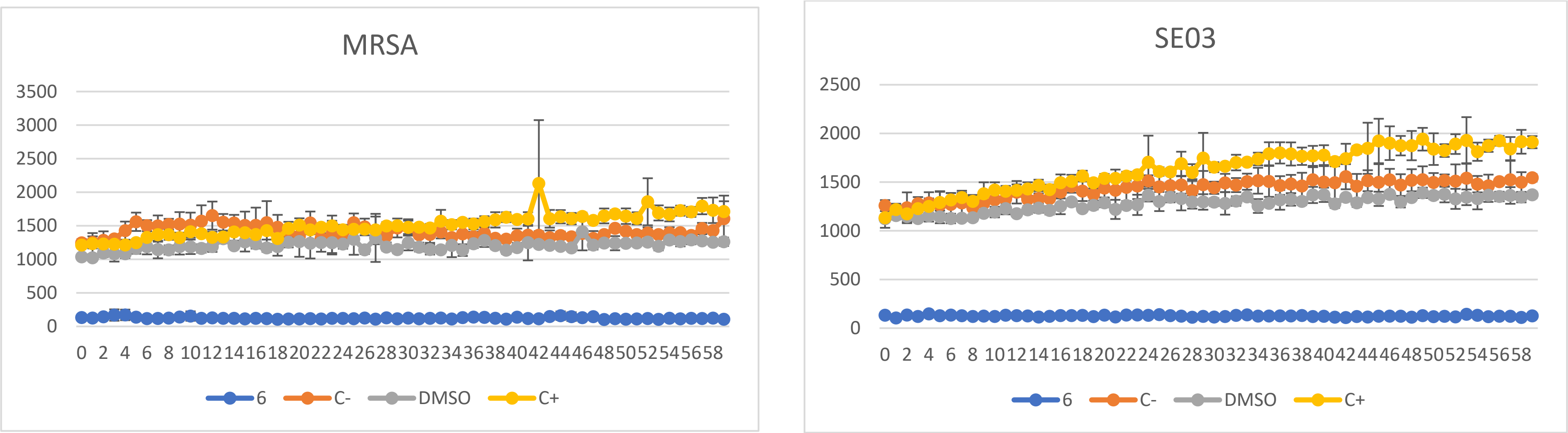

Figure S6. Fluorescence curves for the EB accumulation assay for compound **6**. Conditions: **6** – 50 µM of compound **6** in a solution of EB in PBS (1 µg/mL); **C-** – Bacteria in a solution of EB in PBS (1 µg/mL); **C+** – 25 µM of reserpine in a solution of EB in PBS (1 µg/mL); **DMSO** – 1% v/v of DMSO in a solution of EB in PBS (1 µg/mL)

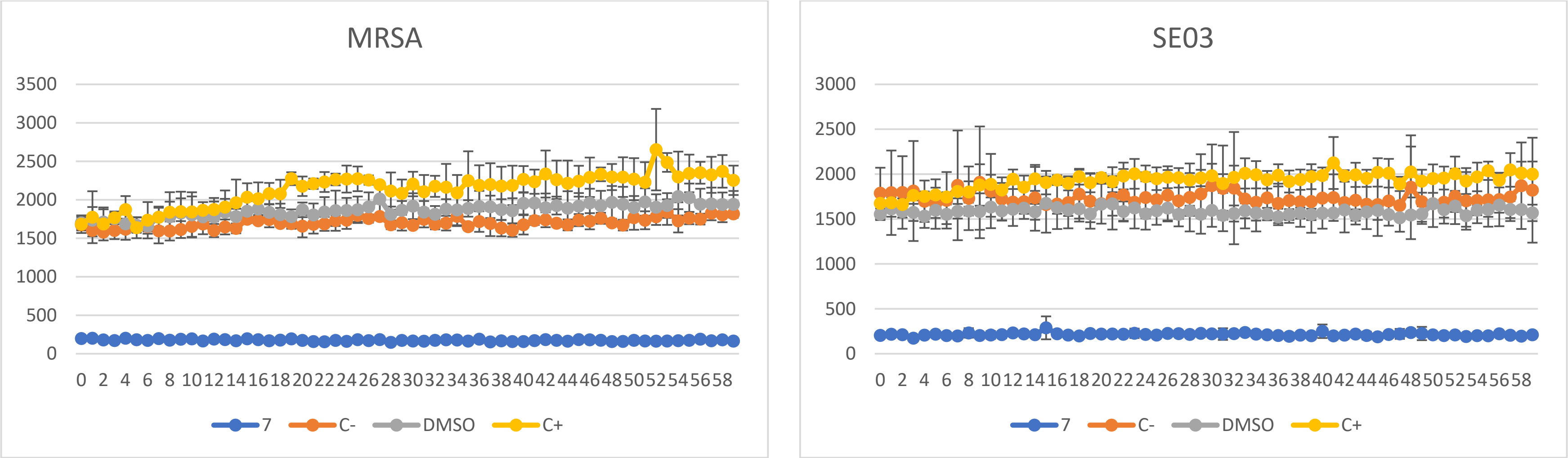

Figure S7. Fluorescence curves for the EB accumulation assay for compound **7**. Conditions: **7** – 50 µM of compound **7** in a solution of EB in PBS (1 µg/mL); **C-** – Bacteria in a solution of EB in PBS (1 µg/mL); **C+** – 25 µM of reserpine in a solution of EB in PBS (1 µg/mL); **DMSO** – 1% v/v of DMSO in a solution of EB in PBS (1 µg/mL)

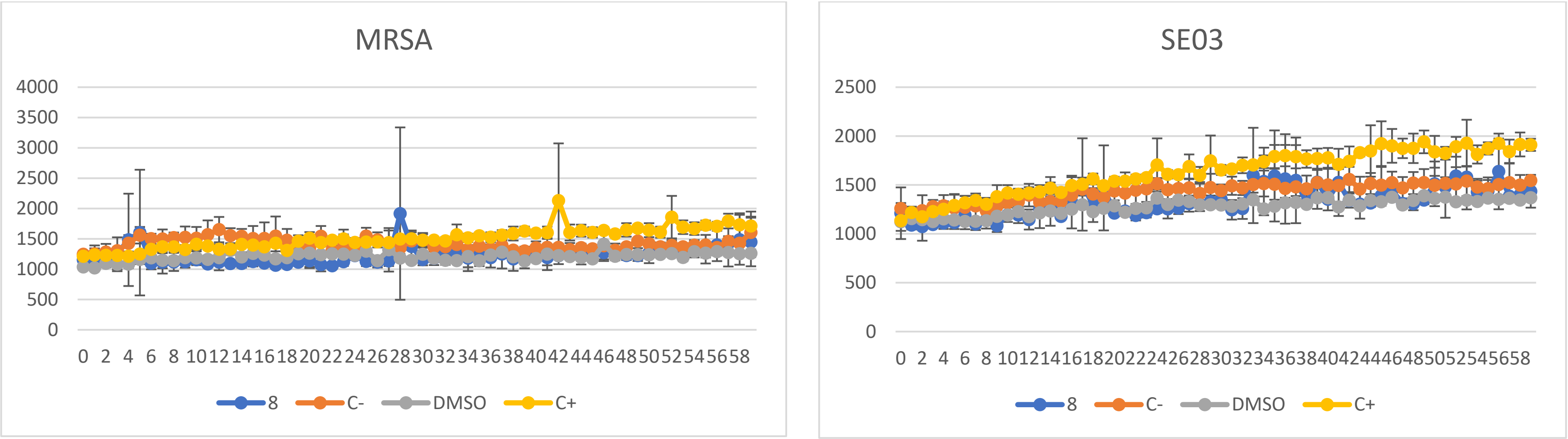

Figure S8. Fluorescence curves for the EB accumulation assay for compound **8**. Conditions: **8** – 50 µM of compound **8** in a solution of EB in PBS (1 µg/mL); **C-** – Bacteria in a solution of EB in PBS (1 µg/mL); **C+** – 25 µM of reserpine in a solution of EB in PBS (1 µg/mL); **DMSO** – 1% v/v of DMSO in a solution of EB in PBS (1 µg/mL)

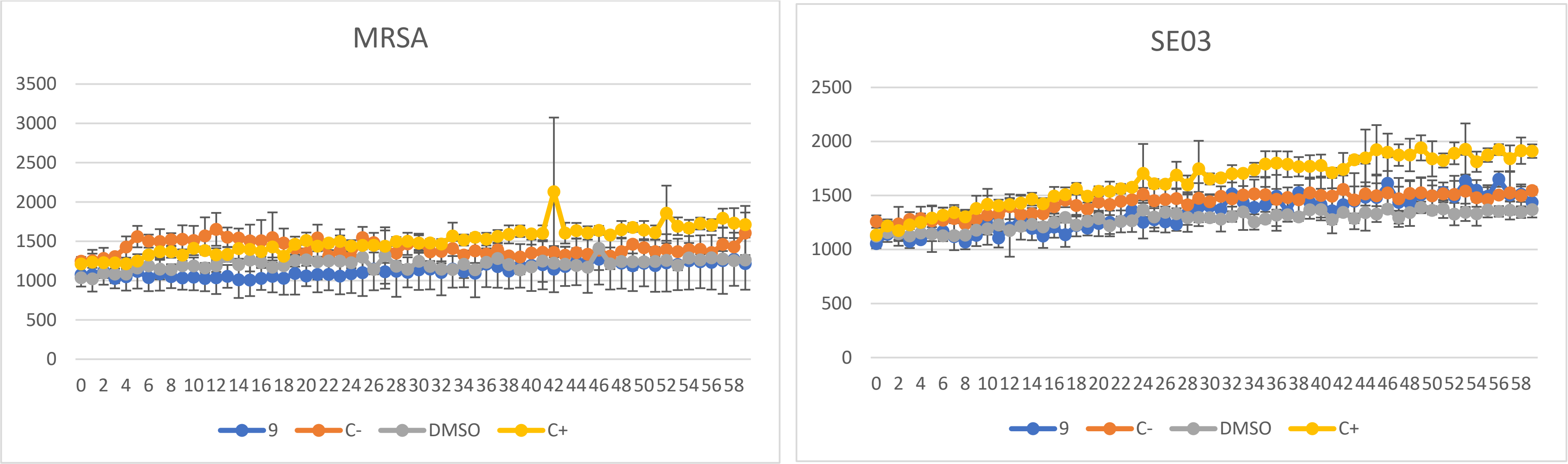

Figure S9. Fluorescence curves for the EB accumulation assay for compound **9**. Conditions: **9** – 50 µM of compound **9** in a solution of EB in PBS (1 µg/mL); **C-** – Bacteria in a solution of EB in PBS (1 µg/mL); **C+** – 25 µM of reserpine in a solution of EB in PBS (1 µg/mL); **DMSO** – 1% v/v of DMSO in a solution of EB in PBS (1 µg/mL)

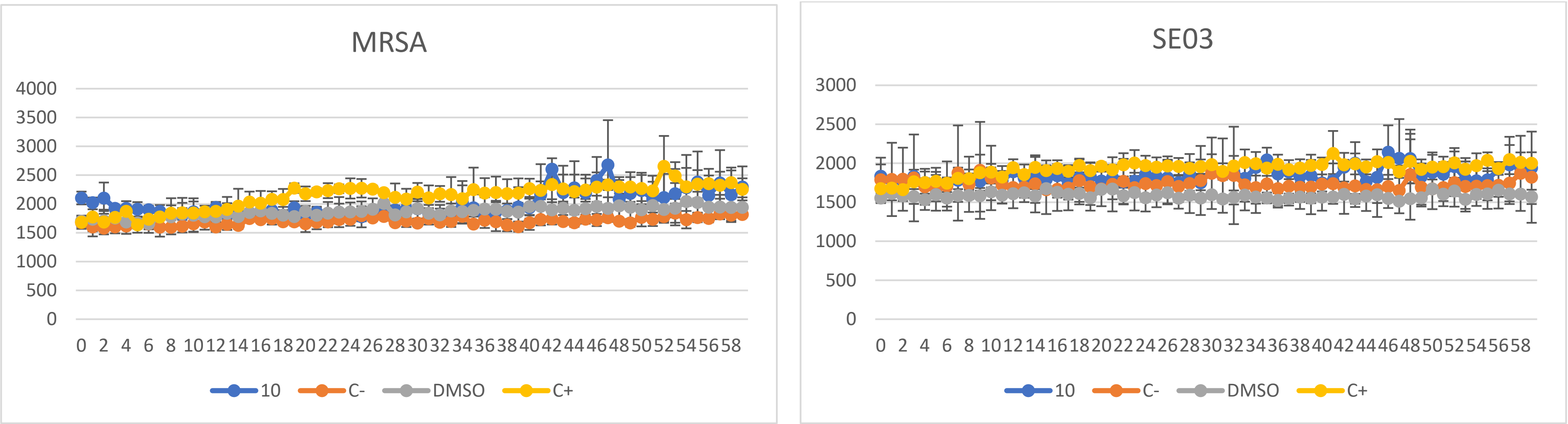

Figure S10. Fluorescence curves for the EB accumulation assay for compound **10**. Conditions: **10** – 50 µM of compound **10** in a solution of EB in PBS (1 µg/mL); **C-** – Bacteria in a solution of EB in PBS (1 µg/mL); **C+** – 25 µM of reserpine in a solution of EB in PBS (1 µg/mL); **DMSO** – 1% v/v of DMSO in a solution of EB in PBS (1 µg/mL)

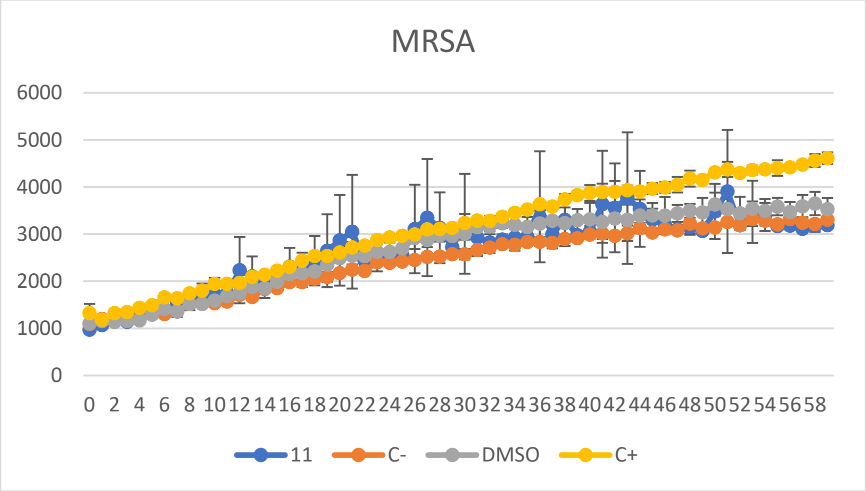

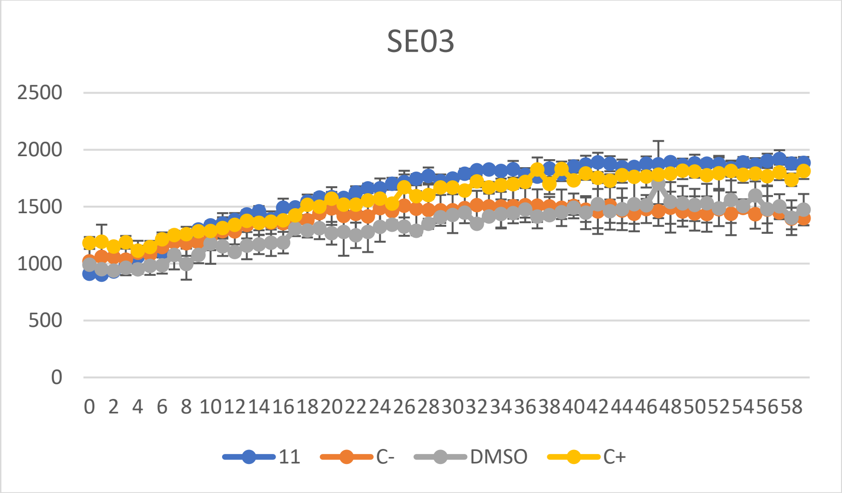

Figure S11. Fluorescence curves for the EB accumulation assay for compound **11**. Conditions: **11** – 50 µM of compound **11** in a solution of EB in PBS (1 µg/mL); **C-** – Bacteria in a solution of EB in PBS (1 µg/mL); **C+** – 25 µM of reserpine in a solution of EB in PBS (1 µg/mL); **DMSO** – 1% v/v of DMSO in a solution of EB in PBS (1 µg/mL)

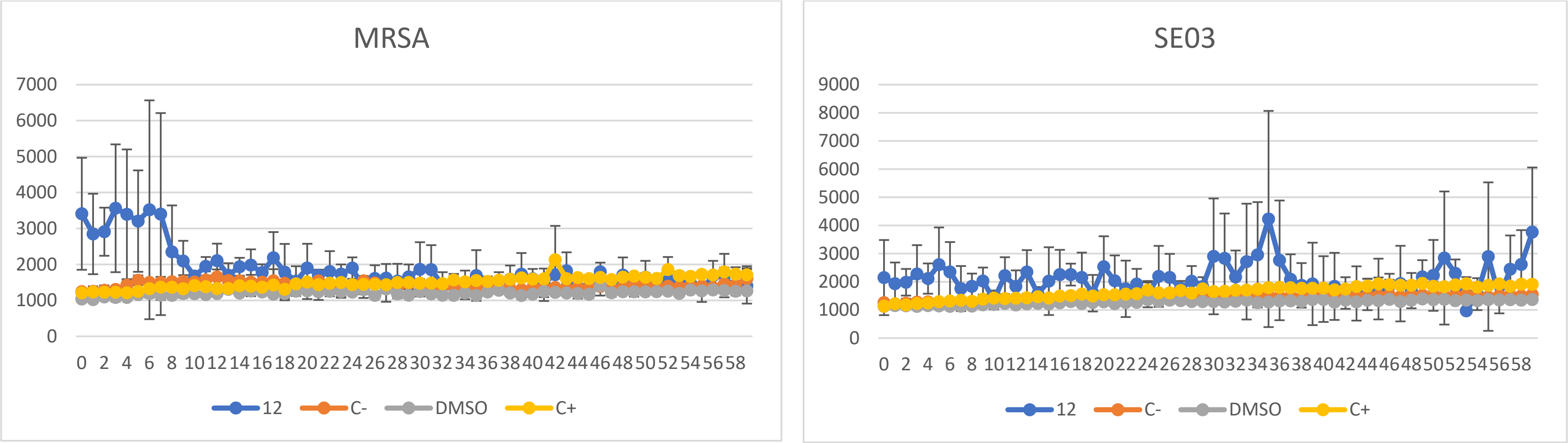

Figure S12. Fluorescence curves for the EB accumulation assay for compound **12**. Conditions: **12** – 50 µM of compound **12** in a solution of EB in PBS (1 µg/mL); **C-** – Bacteria in a solution of EB in PBS (1 µg/mL); **C+** – 25 µM of reserpine in a solution of EB in PBS (1 µg/mL); **DMSO** – 1% v/v of DMSO in a solution of EB in PBS (1 µg/mL)

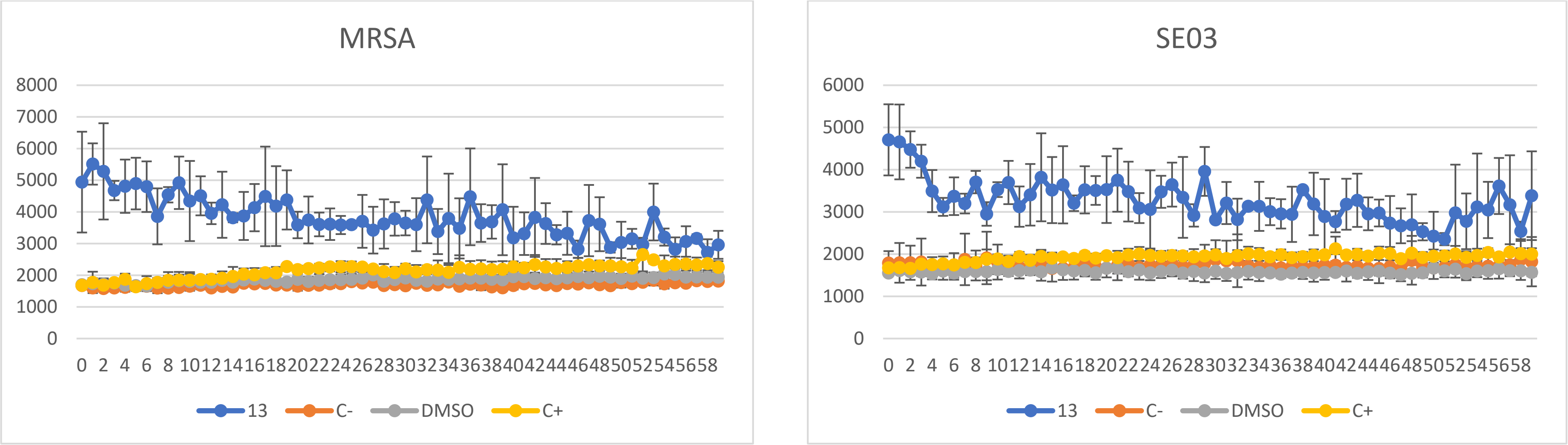

Figure S13. Fluorescence curves for the EB accumulation assay for compound **13**. Conditions: **13** – 50 µM of compound **13** in a solution of EB in PBS (1 µg/mL); **C-** – Bacteria in a solution of EB in PBS (1 µg/mL); **C+** – 25 µM of reserpine in a solution of EB in PBS (1 µg/mL); **DMSO** – 1% v/v of DMSO in a solution of EB in PBS (1 µg/mL)

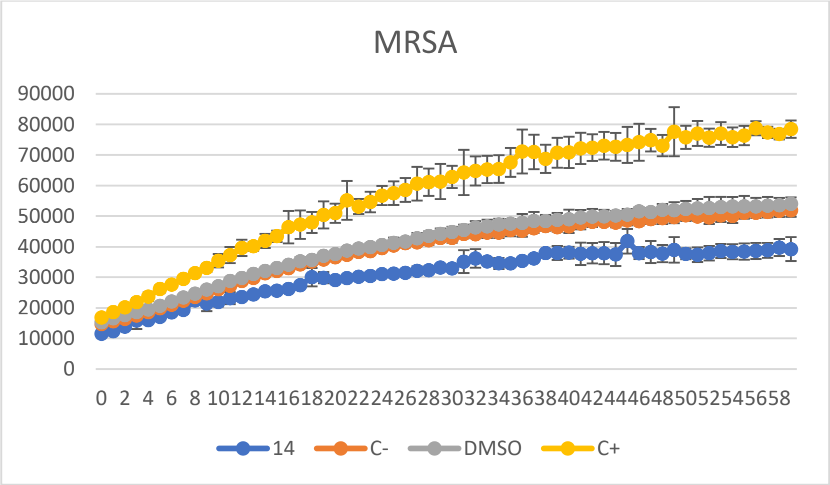

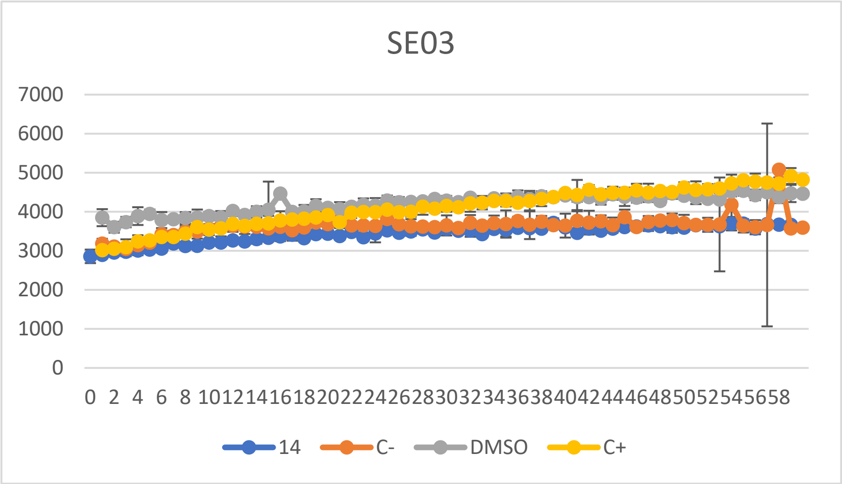

Figure S14. Fluorescence curves for the EB accumulation assay for compound **14**. Conditions: **14** – 50 μM of compound **14** in a solution of EB in PBS (1 μg/mL); **C-** – Bacteria in a solution of EB in PBS (1 μg/mL); **C+** – 25 μM of reserpine in a solution of EB in PBS (1 μg/mL); **DMSO** – 1% v/v of DMSO in a solution of EB in PBS (1 μg/mL)

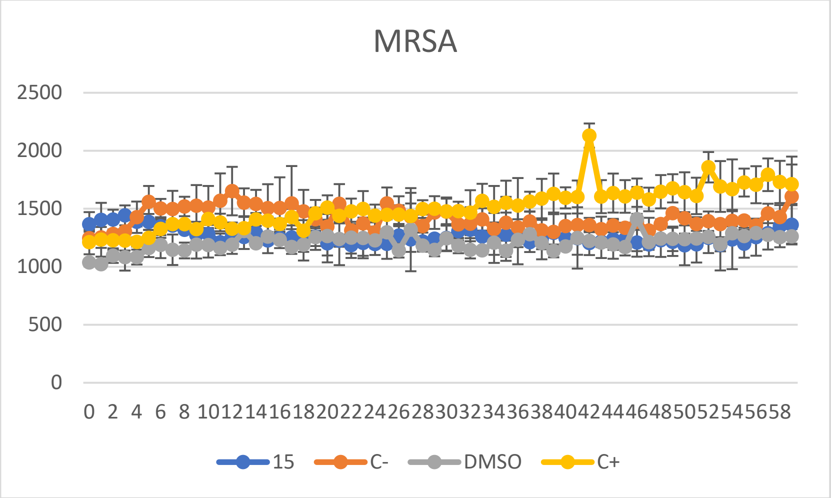

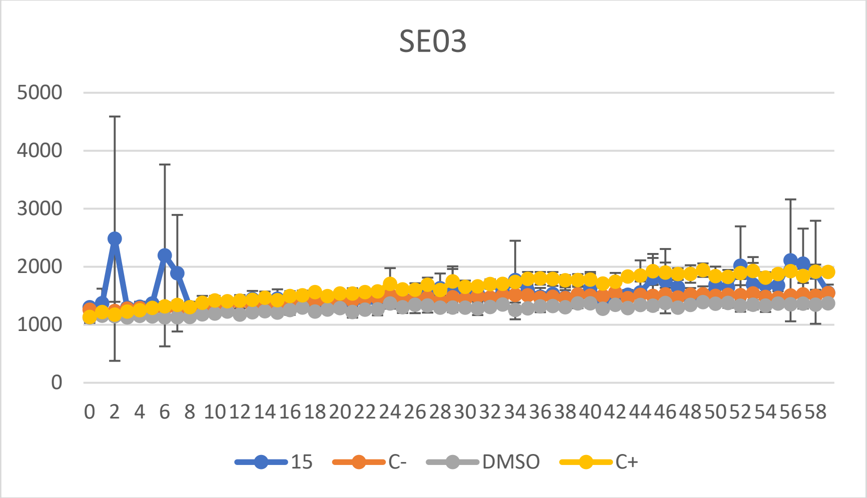

Figure S15. Fluorescence curves for the EB accumulation assay for compound **15**. Conditions: **15** – 50 µM of compound **15** in a solution of EB in PBS (1 µg/mL); **C-** – Bacteria in a solution of EB in PBS (1 µg/mL); **C+** – 25 µM of reserpine in a solution of EB in PBS (1 µg/mL); **DMSO** – 1% v/v of DMSO in a solution of EB in PBS (1 µg/mL)

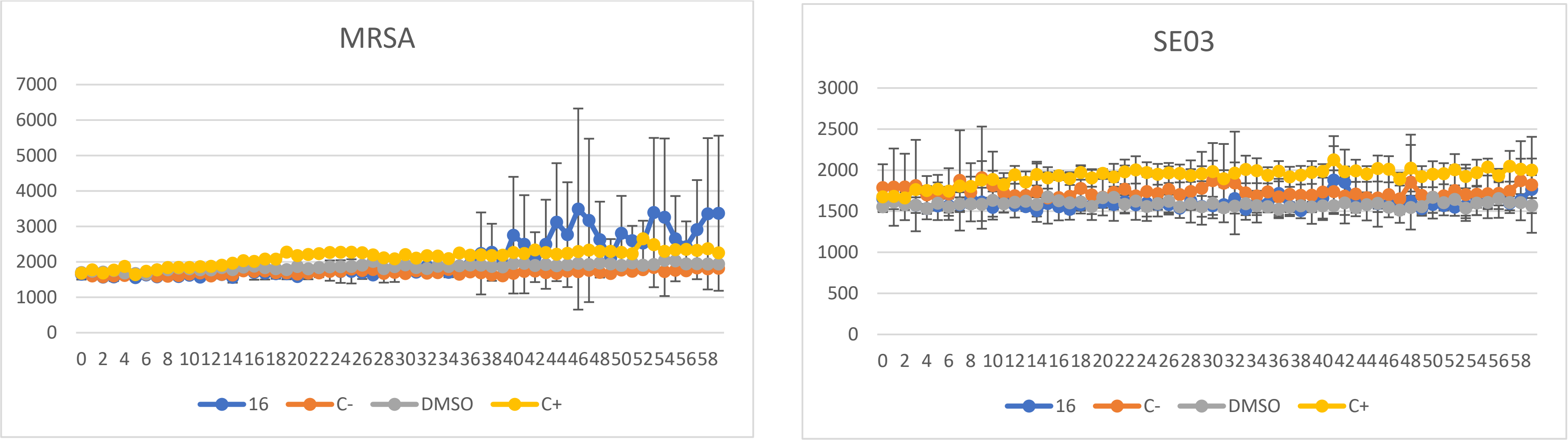

Figure S16. Fluorescence curves for the EB accumulation assay for compound **16**. Conditions: **16** – 50 µM of compound **16** in a solution of EB in PBS (1 µg/mL); **C-** – Bacteria in a solution of EB in PBS (1 µg/mL); **C+** – 25 µM of reserpine in a solution of EB in PBS (1 µg/mL); **DMSO** – 1% v/v of DMSO in a solution of EB in PBS (1 µg/mL)

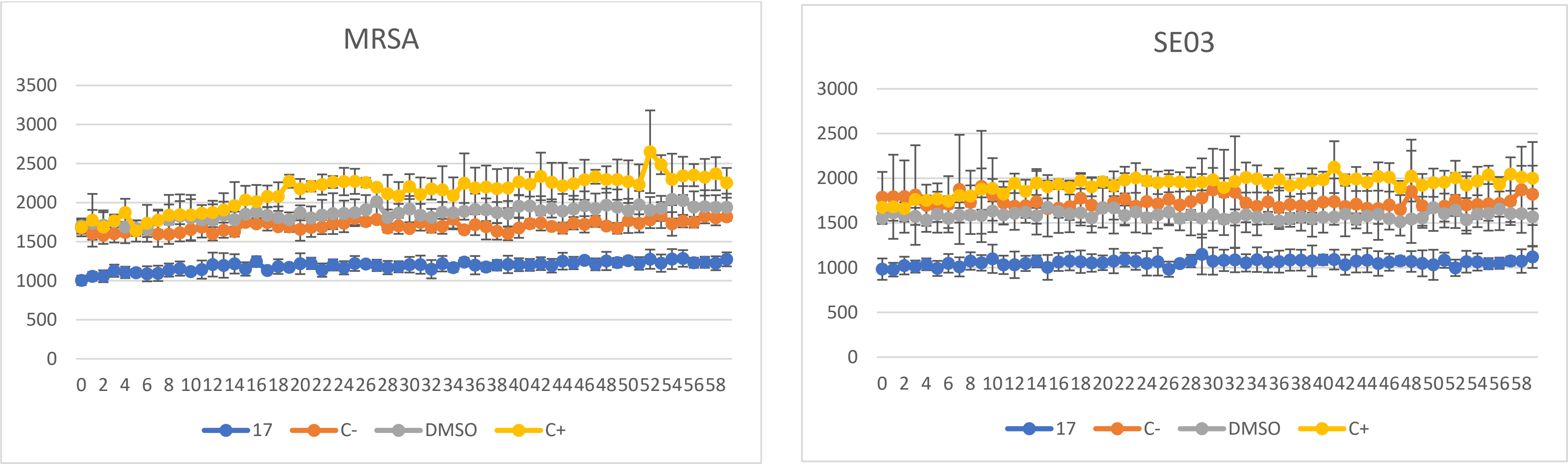

Figure S17. Fluorescence curves for the EB accumulation assay for compound **17**. Conditions: **17** – 50 µM of compound **17** in a solution of EB in PBS (1 µg/mL); **C-** – Bacteria in a solution of EB in PBS (1 µg/mL); **C+** – 25 µM of reserpine in a solution of EB in PBS (1 µg/mL); **DMSO** – 1% v/v of DMSO in a solution of EB in PBS (1 µg/mL)

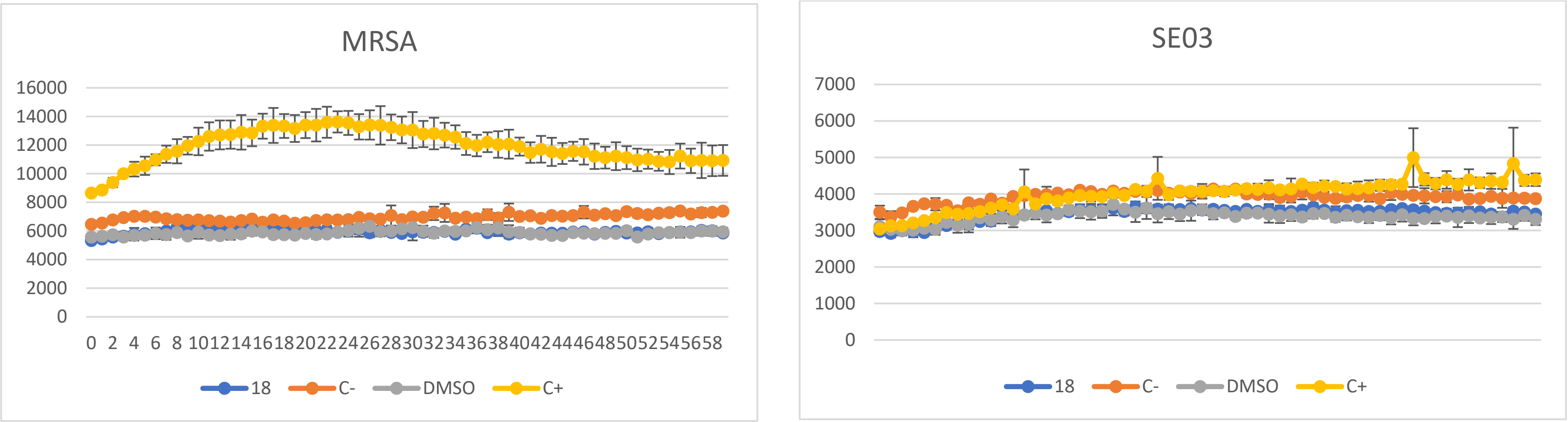

Figure S18. Fluorescence curves for the EB accumulation assay for compound **18**. Conditions: **18** – 50 µM of compound **18** in a solution of EB in PBS (1 µg/mL); **C-** – Bacteria in a solution of EB in PBS (1 µg/mL); **C+** – 25 µM of reserpine in a solution of EB in PBS (1 µg/mL); **DMSO** – 1% v/v of DMSO in a solution of EB in PBS (1 µg/mL)

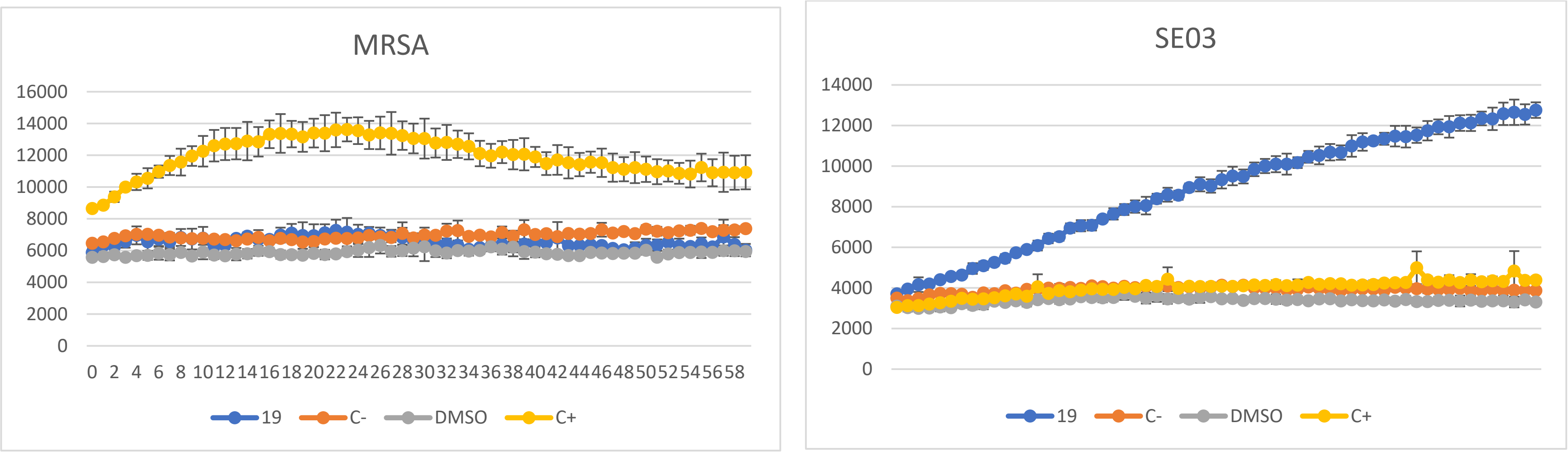

Figure S19. Fluorescence curves for the EB accumulation assay for compound **19**. Conditions: **19** – 50 µM of compound **19** in a solution of EB in PBS (1 µg/mL); **C-** – Bacteria in a solution of EB in PBS (1 µg/mL); **C+** – 25 µM of reserpine in a solution of EB in PBS (1 µg/mL); **DMSO** – 1% v/v of DMSO in a solution of EB in PBS (1 µg/mL)

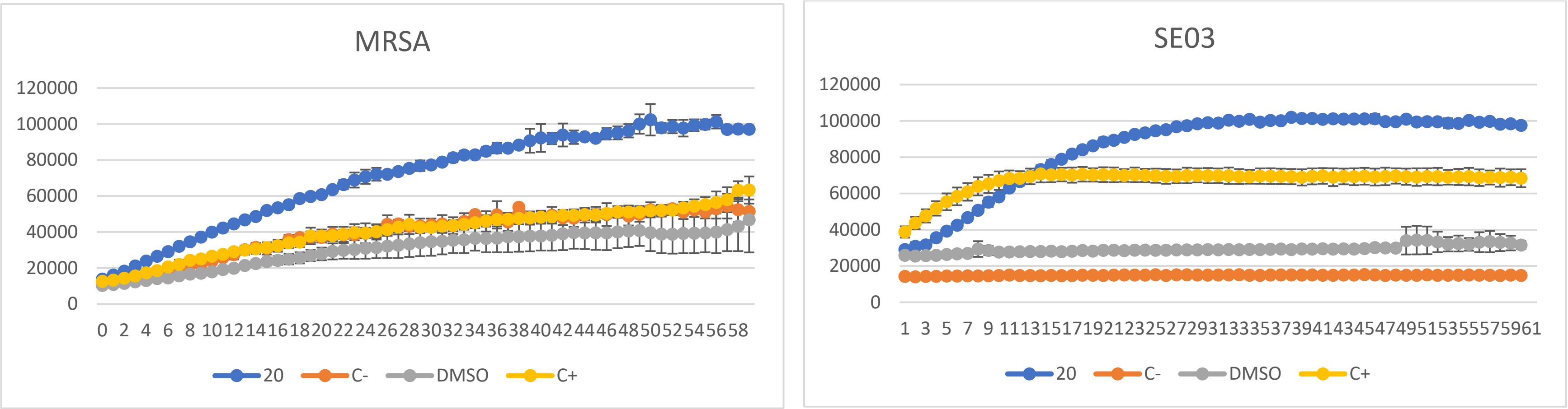

Figure S20. Fluorescence curves for the EB accumulation assay for compound **20**. Conditions: **20** – 50 µM of compound **20** in a solution of EB in PBS (1 µg/mL); **C-** – Bacteria in a solution of EB in PBS (1 µg/mL); **C+** – 25 µM of reserpine in a solution of EB in PBS (1 µg/mL); **DMSO** – 1% v/v of DMSO in a solution of EB in PBS (1 µg/mL)

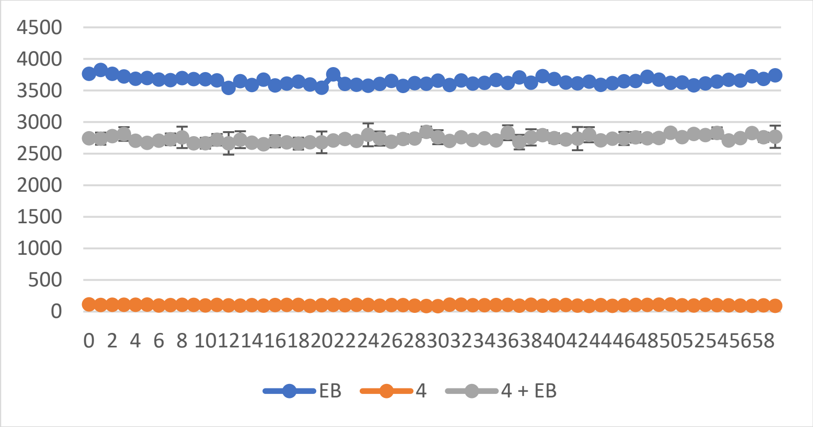

Figure S21. Fluorescence curves for compound **4**. Conditions: **4 + EB**– 50 µM of compound **4** in a solution of EB in PBS (1 µg/mL); **4** – 50 µM of compound **4** in PBS; **EB** – Solution of EB in PBS (1 µg/mL)

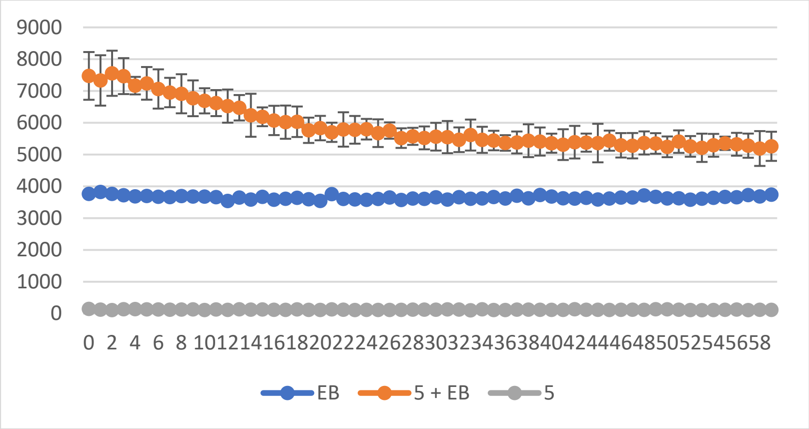

Figure S22. Fluorescence curves for compound **5**. Conditions: **5 + EB**– 50 µM of compound **5** in a solution of EB in PBS (1 µg/mL); **5** – 50 µM of compound **5** in PBS; **EB** – Solution of EB in PBS (1 µg/mL)

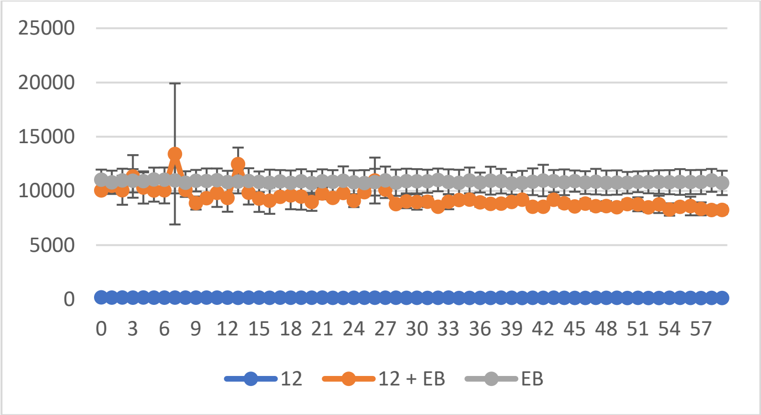

Figure S23. Fluorescence curves for compound **12**. Conditions: **12 + EB**– 50 µM of compound **12** in a solution of EB in PBS (1 µg/mL); **12** – 50 µM of compound **12** in PBS; **EB** – Solution of EB in PBS (1 µg/mL).

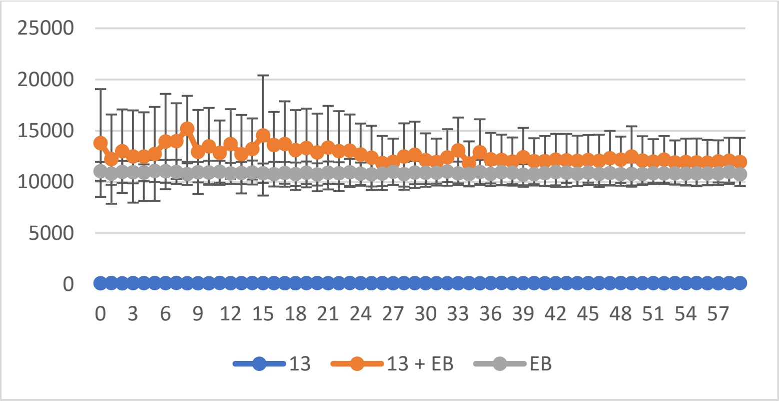

Figure S24. Fluorescence curves for compound **13**. Conditions: **13 + EB**– 50 µM of compound **13** in a solution of EB in PBS (1 µg/mL); **13** – 50 µM of compound **13** in PBS; **EB** – Solution of EB in PBS (1 µg/mL).

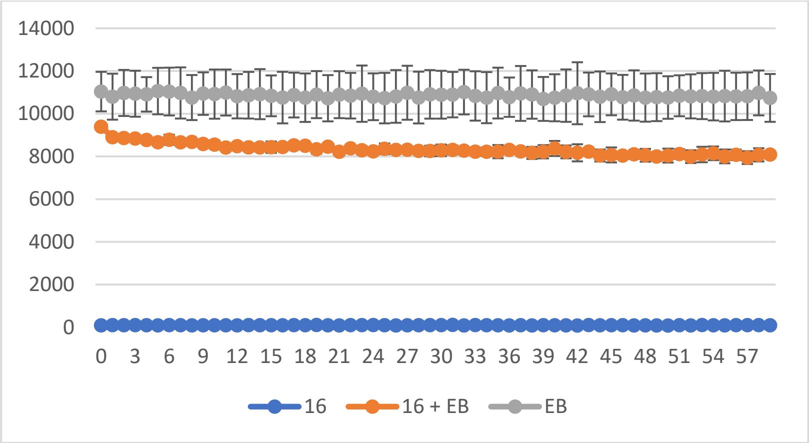

Figure S25. Fluorescence curves for compound **16**. Conditions: **16 + EB**– 50 µM of compound **16** in a solution of EB in PBS (1 µg/mL); **16** – 50 µM of compound **16** in PBS; **EB** – Solution of EB in PBS (1 µg/mL).
